# Development and Implementation of an OSCE for Formative Assessment of Core Clinical Skills in Internal Medicine Interns

**DOI:** 10.15766/mep_2374-8265.11576

**Published:** 2026-02-20

**Authors:** Alexandra Wick, Samantha Murray-Bainer, Jessica Tischendorf, Mazen Almasry, Andrew Coyle, Maryam Zamanian

**Affiliations:** 1 Assistant Professor, Division of Hospital Medicine, Department of Medicine, University of Wisconsin School of Medicine and Public Health; 2 Assistant Professor (CHS), Division of Hospital Medicine, Department of Medicine, University of Wisconsin School of Medicine and Public Health; 3 Assistant Professor, Division of Infectious Disease, Department of Medicine, University of Wisconsin School of Medicine and Public Health; 4 Chief Resident, Department of Medicine, University of Wisconsin School of Medicine and Public Health; 5 Associate Professor, Division of General Internal Medicine, Department of Medicine, University of Wisconsin School of Medicine and Public Health

**Keywords:** OSCE, Residency, Communication Skills, Feedback, Internal Medicine, Clinical Skills Assessment/OSCEs, Simulation, Standardized Patient

## Abstract

**Introduction:**

Direct observation and feedback are essential for developing core intern skills including communicating with consultants, obtaining informed consent, providing handoffs, and responding to nursing concerns. Early in training, the amount of direct observation of these skills varies and is often limited.

**Methods:**

We created a 2-hour, 4-station OSCE for internal medicine interns after completion of inpatient rotations. Stations included (1) calling a consult, (2) obtaining informed consent, (3) providing sign-out, and (4) triaging and responding to nursing pages. Each station was scored using a checklist, and interns received immediate formative feedback from faculty after completing each station. Interns completed pre- and postsession surveys (ratings on 5-point scale).

**Results:**

Thirty-five interns completed the OSCE. The survey response rate was 93%. Checklist data revealed areas for improvement in all stations. Interns reported highly valuing the OSCE, resulting in increased satisfaction (pre- to postsession) with observation and feedback in calling consults (mean 3.69 to 4.15, *p* < .01), obtaining informed consent (mean 3.0 to 4.0, *p* < .01), and responding to nursing pages (mean 3.41 to 4.06, *p* < .01), and increased confidence in obtaining informed consent (mean 3.38 to 4.09, *p* < .01), providing sign-out (mean 3.5 to 4.06, *p* < .01), and responding to nursing pages (mean 3.75 to 4.18, *p* < .01).

**Discussion:**

The OSCE effectively provided direct observation and formative feedback to interns on core clinical skills. It is applicable for internal medicine residency programs to assess interns’ skills and identify areas for improvement early in their training.

## Educational Objectives

By the end of this activity, learners will be able to:

1. Request specialty consultation using a structured format.
2. Identify the critical elements of the informed consent process for a blood transfusion.
3. Apply the I-PASS (Illness Severity, Patient Summary, Action List, Situational Awareness and Contingency Planning, Synthesis by Receiver) handoff framework when providing verbal sign-out on patients.
4. Demonstrate knowledge and skills in triaging and responding to common pages on inpatient medicine wards.

## Introduction

Internal medicine interns are the primary contact for patients, nursing staff, and consultants, and must learn rapidly to prioritize tasks effectively. Many medical schools and residency programs have implemented preparatory curricula to ease the transition from undergraduate to graduate medical education, but the content and focus of these courses are variable.^1–4^ Further, the opportunity for direct, observed feedback of these skills during the intern year may be inconsistent and limited owing to competing clinical priorities, lack of time, and faculty availability.^5^ Therefore, competency is often assumed to be obtained merely through the passage of time.

We identified 4 core skills performed by internal medicine interns that would benefit from direct observation and feedback: (1) calling a consult, (2) obtaining informed consent, (3) providing sign-out, and (4) triaging and responding to nursing pages. Selection of these skills was informed by a national survey of program directors, recommendations from the Alliance for Academic Internal Medicine, and a previous survey of internal medicine residents.^1,6,7^ Several simulation-based curricula have been developed to help medical students or interns learn essential skills in triaging and responding to pages from nurses,^8–10^ providing sign-out to colleagues,^11–14^ calling consultants,^8,15,16^ and obtaining informed consent.^17^ The use of OSCEs to assess these skills is more limited.

Formative assessment with OSCEs has been described in evaluating communication skills among medical students in internal medicine and general surgery,^18,19^ and procedural skills among emergency medicine residents.^20^ Two institutions have described broader OSCEs occurring during orientation to assess the preparedness of incoming interns in several skills.^21,22^ An OSCE during orientation assesses skills acquired in medical school, ensuring baseline ability before entering clinical rotations; however, even in a safe learning climate, this very early baseline assessment can feel evaluative. Assessing core intern skills with an OSCE after they have acquired clinical experience in their role is less well-described. We sought to contribute to the existing literature by developing an OSCE for formative assessment of key intern skills, administered after they had completed at least one inpatient rotation. We hypothesized that this timing would allow for a fuller assessment of their abilities and yield more meaningful feedback on how to further develop their skills, independent of their baseline.

To our knowledge, this is the first OSCE specifically for internal medicine interns, occurring early in training but after relevant clinical experiences.^18–23^ This approach adds to the existing literature in allowing for evaluation of interns’ trajectories in developing these important skills, after they have had the opportunity to practice them during a rotation. It also allowed us to increase the complexity of clinical scenarios. Faculty observations during the OSCE may facilitate the earlier identification of those who may require additional support to be successful, while also providing valuable insights to individual residents regarding areas for growth.

## Methods

We piloted the OSCE on 2 dates in October 2024 for all categorical and primary care internal medicine interns at the University of Wisconsin Hospitals and Clinics, a mid-sized residency program at an academic tertiary care center. During orientation in June 2024, interns received education on the I-PASS (Illness Severity, Patient Summary, Action List, Situational Awareness and Contingency Planning, Synthesis by Receiver) curriculum,^11,12^ basic organizational systems, and calling consults. Prior to the OSCE, all participating interns had rotated on at least one inpatient service where all these skills would have been practiced.

### Development

Our team consisted of a hospitalist with simulation expertise, clinical coaches (2 hospitalists, 1 infectious disease specialist), a chief resident, a senior resident, and the internal medicine residency program director. Faculty physicians wrote the scenarios, and the chief resident and senior resident reviewed them to ensure fidelity to the intern experience. This study was considered to be program evaluation by the Institutional Review Board at the University of Wisconsin-Madison.

### Equipment/Environment for the OSCE

The OSCE was held in the University of Wisconsin-Madison Clinical Teaching and Assessment Center (CTAC). Each 2-hour OSCE utilized 8 clinic rooms, with 2 sets of Stations A–D running simultaneously, to accommodate 7–10 interns per session. We ran the OSCE 4 times to accommodate all 35 interns. The personnel and equipment needed to set up each station are detailed in Table 1 and the Figure. Each station incorporated an embedded participant, an individual trained to play a role in a simulation encounter to guide the scenario, and a faculty observer. The embedded participants participated in person or via telephone. Two stations required a working telephone. One station required a pager. Three stations contained a computer, with case documents uploaded to the desktop, although physical copies would be an adequate substitute.

**Table 1.**
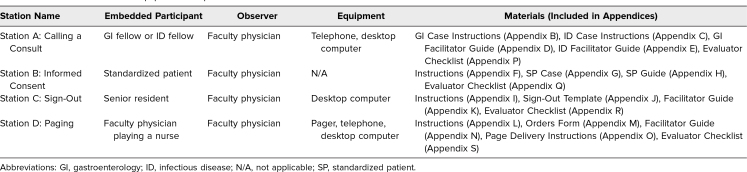
Station Personnel and Equipment Setup for the OSCE on Formative Assessment of Core Clinical Skills in Internal Medicine Interns

**Figure.**
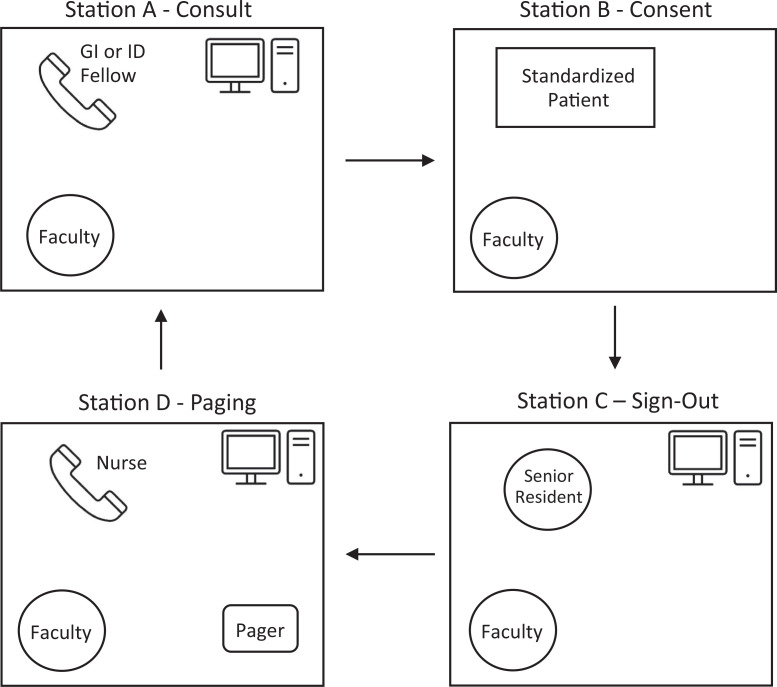
Schematic of the layout of the OSCE on formative assessment of core clinical skills in internal medicine interns. The interns rotate through 4 stations, Stations A–D. Each station lasts 20 minutes, with 15 minutes to complete the tasks and 5 minutes for feedback. Four interns complete all 4 stations in 90 minutes. Abbreviations: GI, gastroenterology; ID, infectious disease.

### Personnel

For each 2-hour OSCE, we scheduled the following people to facilitate:

• Eight faculty physicians as observers (1 per room for Stations A–D)
• One gastroenterology (GI) fellow physician and 1 infectious disease (ID) fellow physician (1 per room for Station A)
• Two standardized patients (SPs; 1 per room for Station B)
• Two senior residents (1 per room for Station C)
• Two faculty physicians or nurse practitioners to play a nurse (1 per room for Station D)
• One coordinator to send pages (1 for both rooms for Station D)

We recruited the faculty physicians, fellows, senior residents, and coordinator from the Department of Medicine and the nurse practitioner from the Division of Hospital Medicine. The CTAC recruited the SPs.

### Implementation

At the beginning of the session, a hospitalist with simulation expertise led a 20-minute prebrief with the interns to review the intent and structure of the OSCE (Appendix A). Each intern rotated through four 20-minute stations (Stations A–D) simulating inpatient medical scenarios. After 15 minutes elapsed in each station, the embedded participant and faculty observer provided 5 minutes of feedback to the intern.

#### Station A—calling a consult

We instructed the interns to read a clinical note and request a consult for a patient. Due to the limited fellow availability, we created 2 different cases, and interns participated in 1 or the other case. The GI case described a patient with choledocholithiasis and acute cholangitis, and interns were instructed to request a consult from GI (Appendix B). The ID case described a patient with *Staphylococcus aureus* bacteremia, and interns were instructed to request an ID consult (Appendix C). The interns used the telephone in the room to call the fellow at a provided number. The GI and ID fellow embedded participants were provided with facilitator guides (Appendix D, Appendix E) to help them probe the intern's understanding of the case, the urgency of the consultation, and their ability to perform closed-loop communication.

#### Station B—obtaining informed consent

We asked the interns to obtain informed consent for a blood transfusion from a patient (Appendix F). The case described a patient with symptomatic anemia due to a gastrointestinal bleed and a drop in hemoglobin below the transfusion threshold (Appendix G). In the room, an SP was dressed in a hospital gown and laid in a hospital bed. We trained the SP using a script that included their medical history, reason for hospitalization, and questions to ask the intern (Appendix H). We provided our institutional blood transfusion consent form for the SP to sign during the scenario.

#### Station C—providing sign-out

We instructed the interns to provide verbal sign-out and generate written sign-out for 2 complex patients. Interns read the clinical notes (Appendix I) and then typed their sign-out into a template mimicking the tool used in the residency program (Appendix J). After 10 minutes had elapsed, a senior resident entered the room, and the intern had 5 minutes to deliver verbal sign-out. We provided a facilitator guide to the senior resident (Appendix K) to prepare for their role. The senior resident and the faculty observer provided feedback only on the verbal sign-out.

#### Station D—responding to pages from nurses

We asked the interns to respond to pages from nurses regarding patients they were covering overnight. We provided the interns with a pager, a written sign-out document containing patient information (Appendix L), and a form to write orders (Appendix M). We asked the interns to triage the pages by priority, and either call back by phone or write orders on the provided form as they deemed appropriate. At the beginning of the station, we sent 3 simultaneous pages: 1 of low priority (request for bowel regimen), 1 of high priority (agitation management), and 1 of medium priority (rash due to vancomycin). After 8 minutes had elapsed, we sent 2 additional simultaneous pages: 1 of low priority (potassium repletion) and 1 of high priority (fever). We created a detailed script for the embedded participant playing the nurse, including case details and questions to ask the intern to probe their clinical reasoning or challenge their plan (Appendix N). In addition, we created instructions for a coordinator who assisted with sending the pages at the correct times (Appendix O).

### Learner Assessment

We developed station-specific checklists for faculty observers to ensure structured feedback on educational objectives. The checklists informed immediate verbal feedback to the intern after the conclusion of a station. We provided detailed instructions to the faculty evaluator for each station, including a request to incorporate verbal feedback from the embedded participant in their written assessment.

#### Station A—calling a consult

We assessed the interns’ ability to request consultation in a structured manner, including the consult question and pertinent patient information. We developed the assessment checklist for this station (Appendix P) using principles from Kessler's 5 C's^15,16^ of Consultation, Contact, Communicate, Core Question, Collaboration, and Closing the Loop. We modified the checklist based on local experience. A global rating scale was included to assess overall communication effectiveness and interpersonal professionalism.

#### Station B—obtaining informed consent

We assessed the interns’ ability to communicate the risks and benefits of blood transfusion to the patient, explore alternatives, and assess patient understanding while obtaining informed consent. We designed the checklist for this station (Appendix Q) in alignment with institutional standards and previous evaluation tools published in *MedEdPORTAL*.^17^

#### Station C—providing sign-out

We assessed the interns’ verbal sign-out skills in a structured manner, utilizing the I-PASS framework^11–13^ and emphasizing relevant patient information and contingency plans. The evaluation instrument for this station (Appendix R) was based on the previously published I-PASS handoff tool,^13^ with slight modifications for this exercise. The checklist only assessed the verbal sign-out component of this station.

#### Station D—responding to pages from nurses

We assessed the interns’ ability to triage pages appropriately according to priority, communicate effectively with the nurses, and use clinical reasoning to address common inpatient medical problems. We developed a detailed checklist (Appendix S) for the cases in this station, with the structure informed by previously published curricula.^9^

### Assessment of the OSCE

We distributed surveys to the interns before and after the OSCE to evaluate the effectiveness of the educational intervention (Appendix T). Interns were queried regarding their satisfaction with the direct observation and feedback and their confidence in performing the 4 core skills, rating their level of agreement or disagreement with statements on a 5-point Likert scale (1 = *strongly disagree*, 2 = *disagree*, 3 = *neutral*, 4 = *agree*, 5 = *strongly agree*). In comparing pre- and postsession mean survey scores, *t* tests were used to determine statistically significant differences. Effect sizes were calculated using Hedges’ *g* (given differences in group size), with effect sizes considered to be small (Hedges’ *g* ∼ 0.2), medium (Hedges’ *g* ∼ 0.5), or large (Hedges’ *g* > 0.8).

## Results

Thirty-five internal medicine interns participated in the OSCE in October of 2024. For Station A, 68.6% of interns stated their rank/service, 73.5% identified the consultant physician's name, 51.4% specified the timeframe for the consultation, and 50% repeated the patient care plan. All other items were marked as completed in more than 80% of assessments. For Station B, more than 80% of interns completed each item, except for discussion of no transfusion as an option (61.7%) and alternatives to transfusion (75.8%). For Station C, 44.4% of interns addressed illness severity completely, 95% provided a patient summary, 78.5% provided an action list, 66.7% addressed situational awareness, and 56.8% ensured synthesis by the receiver. For Station D, more than 80% of participants achieved all metrics, except for stating plans to evaluate patients when appropriate (70.6%), explaining thought process (71.4%), asking the nurse for questions (39.3%), and using closed-loop communication (40%). In every station, more than 70% of faculty assessments included a written comment identifying an area for improvement.

Thirty-two residents (91%) completed the presession survey, and 33 (94%) completed the postsession survey, for a total response rate of 93% (65/70). All surveys were at least partially completed; however, 5 were excluded from our analysis due to incomplete or indecipherable responses.

Perception of adequacy of the direct observation and feedback and interns’ self-reported confidence in performing these skills improved after the OSCE (Table 2). The interns’ perceptions of receiving sufficient direct observation and feedback significantly increased from pre- to postsession, in calling a consult (mean 3.69 to 4.15, *p* < .01), obtaining informed consent (mean 3.0 to 4.0, *p* < .01), and responding to nursing pages (mean 3.41 to 4.06, *p* < .01). There was no significant difference in interns’ perceptions of the adequacy of observation and feedback on providing sign-out (*p* = .28). The largest effect size was observed for obtaining informed consent (Hedges’ *g* 1.261).

**Table 2.**
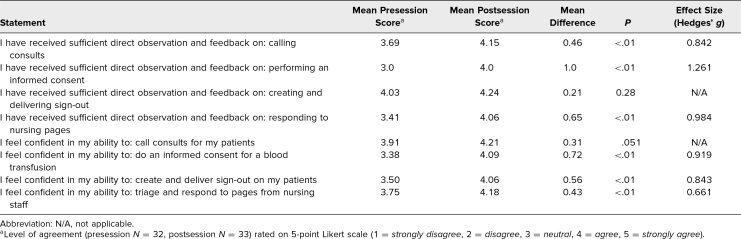
Intern Satisfaction and Confidence Ratings Before and After the OSCE on Formative Assessment of Core Clinical Skills in Internal Medicine Interns

Following the OSCE, self-reported confidence significantly increased for obtaining informed consent (mean 3.38 to 4.09, *p* < .01), providing sign-out (mean 3.5 to 4.06, *p* < .01), and responding to nursing pages (mean 3.75 to 4.18, *p* < .01). Interns’ confidence post-OSCE did not significantly increase for calling a consult (*p* = .051). Again, the largest effect size was seen for obtaining informed consent (Hedges’ *g* 0.919).

After the OSCE, 97% of interns *agreed* or *strongly agreed* that the exercise was valuable, and 94% *agreed* or *strongly agreed* that it should be continued for future years. Twenty-four residents provided written comments (Table 3), which were generally positive, including satisfaction with the realism of cases, the paging station, and the usefulness of feedback received. A few interns requested that the OSCE be offered earlier in the year, with a suggested timing of 1–2 months into training. Some felt that the session being designated as an OSCE was anxiety-inducing and did not reflect the nature of the experience, requesting it be renamed.

**Table 3.**
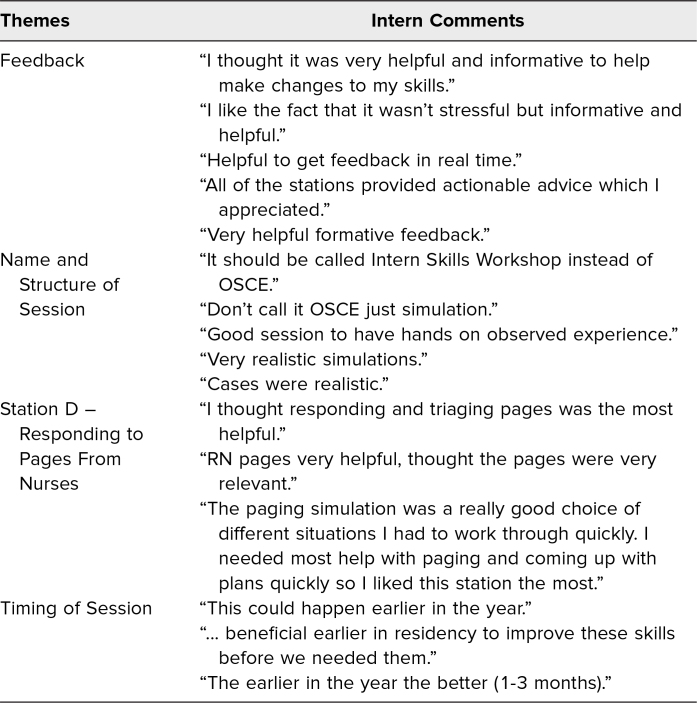
Intern Comments From Post-OSCE Surveys

## Discussion

To address the need for increased direct observation and feedback for core intern skills, we designed a 4-station OSCE that assessed interns’ abilities in calling a consult, obtaining informed consent, providing sign-out, and addressing pages from nurses. This OSCE actively engaged interns through simulated encounters followed by immediate, formative feedback. The unique timing and comprehensive nature of this OSCE allowed cases of increased complexity, representative of the patients cared for by internal medicine interns.

### Evaluation of Work and Reflection of Results

There was a high degree of acceptance of the OSCE, with more than 90% of interns reporting it as valuable and recommending that we continue it. Interns were satisfied with the realism of the cases, valued the opportunity to practice these skills, and appreciated the feedback they received.

Evaluation of the faculty assessments showed that most interns were competent in these core skills, but areas for improvement were identified for all stations. For calling a consult, the C's that were least discussed included defining a core question and closing the loop. For obtaining informed consent, all the interns discussed the risks of blood transfusion, but fewer discussed a plan if transfusion was not pursued. When providing sign-out, almost all interns provided a patient summary but fewer than 50% addressed illness severity completely. Fewer than two-thirds of interns addressed situational awareness or ensured synthesis by the receiver. For the paging station, the most missed components included asking the nurse for questions and using closed-loop communication. Despite interns receiving formal education on these skills during orientation and practicing them during an inpatient rotation, we identified performance gaps, and faculty delivered targeted, actionable feedback.

Participants reported modest satisfaction with feedback on core skills 3–4 months into their intern year, with lower satisfaction noted for calling consults, obtaining informed consent, and responding to nursing pages compared to providing sign-out. This may be related to our institutional culture, in which senior residents and interns sign out together, leading to frequent direct observation. Following the OSCE, intern perception of adequacy of observation and feedback improved for 3 of 4 skills (all but sign-out). This suggests that even a small amount of focused, direct observation and feedback can improve satisfaction with feedback. Interns felt more confident in performing 3 of 4 skills after the OSCE, with the fourth skill (calling consults) having a strong trend toward benefit. The lack of significant change in skills for calling consults may be related to the interns having higher self-assessed confidence prior to the OSCE, due to experience gained during medical school or in the first few months of residency. Additionally, results could have been influenced by heterogeneity introduced by the 2 different cases for Station A. Due to survey anonymity, we cannot stratify the results by an individual participant's case session.

### Reflections on Development and Implementation

A strength of this study was our high survey completion rate. Universal participation from our intern class was also a strength, as it represented all learners in the cohort and eliminated the potential for volunteer bias. Protecting the interns’ time was a challenge, as well as scheduling volunteer faculty, fellows, and senior residents. This effort required months of advanced planning involving the assistance of chief residents to provide clinical coverage for interns as needed. Remote involvement of embedded participants in Stations A and D allowed for flexibility in scheduling. While a recorded session would require fewer synchronous resources, we believe that incorporating immediate feedback from faculty was essential, as feedback timing is a commonly included element in feedback best practices.^24^ To overcome challenges recruiting fellows from the same subspecialty to volunteer simultaneously, we created 2 different cases for Station A. We feel that the fellows increased realism of the simulation, but any internal medicine faculty could play the role using the detailed facilitator guides.

We learned that the 15 minutes allotted for the sign-out station were insufficient for interns to complete verbal and written sign-out for 2 cases. We found that the observational data from a single case were sufficient to give meaningful feedback. Several interns suggested scheduling the session earlier in the year, but still after some clinical experience. We believe they suggested this because they found the feedback beneficial and would help them perform these skills better as early interns.

### Limitations

We identified several limitations in our OSCE. First, this OSCE was developed and implemented at a single institution. This could potentially limit applicability to residencies similar to ours. Second, using paper assessments led to the omission of some survey data due to indecipherable responses, which may have influenced our results. Last, we assessed the learners with immediate pre- and postsession surveys, which can be expected to show improvement due to the exposure itself. We feel this design was necessary to connect educational gains directly to the intervention. Given the use of these skills in nearly all internal medicine rotations, any longer-term gains in these skills would be difficult to directly attribute to the OSCE and may be influenced by maturation bias.

### Future Directions

We believe that this OSCE is effective as a stand-alone educational intervention. In future studies, we hope to show that integrating OSCE results into a coaching or mentoring program will help target coaching on specific clinical skills and improve performance in the long term. We expect this process may identify interns struggling with core clinical skills earlier than might otherwise come to the program's attention, allowing for early coaching intervention. In future iterations of this OSCE, we will build in time for self-reflection and encourage interns to set goals related to clinical skills in preparation for discussion with their coach.

## Appendices


Prebrief Guide.docxStation A - GI Case Instructions.docxStation A - ID Case Instructions.docxStation A - GI Facilitator Guide.docxStation A - ID Facilitator Guide.docxStation B - Instructions.docxStation B - SP Case.docxStation B - SP Guide.docxStation C - Instructions.docxStation C - Sign-Out Template.docxStation C - Facilitator Guide.docxStation D - Instructions.docxStation D - Orders Form.docxStation D - Facilitator Guide.docxStation D - Page Delivery Instructions.docxStation A - Evaluator Checklist.docxStation B - Evaluator Checklist.docxStation C - Evaluator Checklist.docxStation D - Evaluator Checklist.docxPre- and Postsurveys.docx
*All appendices are peer reviewed as integral parts of the Original Publication.*
