# Supplementary material for: Development and Implementation of an OSCE for Formative Assessment of Core Clinical Skills in Internal Medicine Interns
Source: MedEdPORTAL. 2026 Feb 20;22:11576. doi: 10.15766/mep_2374-8265.11576 (PMC12920606; doi:10.15766/mep_2374-8265.11576)
Supplement: Supplementary file 1 — Prebrief Guide.docxStation A - GI Case Instructions.docxStation A - ID Case Instructions.docxStation A - GI Facilitator Guide.docxStation A - ID Facilitator Guide.docxStation B - Instructions.docxStation B - SP Case.docxStation B - SP Guide.docxStation C - Instructions.docxStation C - Sign-Out Template.docxStation C - Facilitator Guide.docxStation D - Instructions.docxStation D - Orders Form.docxStation D - Facilitator Guide.docxStation D - Page Delivery Instructions.docxStation A - Evaluator Checklist.docxStation B - Evaluator Checklist.docxStation C - Evaluator Checklist.docxStation D - Evaluator Checklist.docxPre- and Postsurveys.docx [file mep_2374-8265.11576-s001.zip › A. Prebrief Guide.docx]

**Appendix A: Intern OSCE Pre-Brief Guide**

**Introduction**

1. Facilitator introductions
2. Check in with how interns are feeling
   1. Validate any performance anxieties
3. Explain the purpose of the OSCE
   1. Provide an opportunity for direct observation of core intern skills they have already been doing in clinical practice
   2. Provide formative feedback to help them improve and identify areas for growth
4. Rules of Simulation
   1. Confidentiality
      1. *“We ask that you keep what happens during the OSCE today confidential. Results will only be shared with your clinical coach and not with the residency leadership. Also, note that these cases may be used again, so please do not give your peers details that might give them an unfair advantage.”*
   2. Basic Assumption
      1. *“We are all under the assumption that everyone participating in the OSCE is intelligent, hardworking, and wants to improve their patient care skills.”*
   3. Fiction Contract
      1. *“We strive to make the scenarios as lifelike as possible, but they cannot be perfect. We ask you to suspend your disbelief and immerse yourself in the simulation to get the most out of this experience today.”*

**Structure of the Session**

1. Rotate through four 20-minute stations
   1. Calling a consult
   2. Obtaining informed consent
   3. Providing sign-out (written and verbal)
   4. Responding to pages from a nurse
2. Will be observed by a faculty member in the room who will provide verbal feedback and complete a checklist
3. Surveys pre- and post-OSCE
