# Supplementary material for: Development and Implementation of an OSCE for Formative Assessment of Core Clinical Skills in Internal Medicine Interns
Source: MedEdPORTAL. 2026 Feb 20;22:11576. doi: 10.15766/mep_2374-8265.11576 (PMC12920606; doi:10.15766/mep_2374-8265.11576)
Supplement: Supplementary file 1 — Prebrief Guide.docxStation A - GI Case Instructions.docxStation A - ID Case Instructions.docxStation A - GI Facilitator Guide.docxStation A - ID Facilitator Guide.docxStation B - Instructions.docxStation B - SP Case.docxStation B - SP Guide.docxStation C - Instructions.docxStation C - Sign-Out Template.docxStation C - Facilitator Guide.docxStation D - Instructions.docxStation D - Orders Form.docxStation D - Facilitator Guide.docxStation D - Page Delivery Instructions.docxStation A - Evaluator Checklist.docxStation B - Evaluator Checklist.docxStation C - Evaluator Checklist.docxStation D - Evaluator Checklist.docxPre- and Postsurveys.docx [file mep_2374-8265.11576-s001.zip › T. Pre- and Postsurveys.docx]

**Appendix T: Intern OSCE Pre- and Post-Surveys**

**Pre-OSCE Survey**

For the following statements, please rate your agreement/disagreement:

|  | Strongly Agree | Agree | Neutral | Disagree | Strongly Disagree |
| --- | --- | --- | --- | --- | --- |
| Thus far during my intern year, I have received sufficient direct observation and feedback on: **responding to nursing pages** |  |  |  |  |  |
| Thus far during my intern year, I have received sufficient direct observation and feedback on: **calling consults** |  |  |  |  |  |
| Thus far during my intern year, I have received sufficient direct observation and feedback on: **performing an informed consent** |  |  |  |  |  |
| Thus far during my intern year, I have received sufficient direct observation and feedback on: **creating and delivering signout** |  |  |  |  |  |
| Direct observation of these skills will be a valuable learning opportunity |  |  |  |  |  |
| I feel confident in my ability to **create and deliver signout on my patients** |  |  |  |  |  |
| I feel confident in my ability to **call consults for my patients** |  |  |  |  |  |
| I feel confident in my ability to **triage and respond to pages from nursing staff** |  |  |  |  |  |
| I feel confident in my ability to do an **informed consent for a blood transfusion** |  |  |  |  |  |

**Post-OSCE Survey**

For the following statements, please rate your agreement/disagreement:

|  | Strongly Agree | Agree | Neutral | Disagree | Strongly Disagree |
| --- | --- | --- | --- | --- | --- |
| Thus far during my intern year, I have received sufficient direct observation and feedback on: **responding to nursing pages** |  |  |  |  |  |
| Thus far during my intern year, I have received sufficient direct observation and feedback on: **calling consults** |  |  |  |  |  |
| Thus far during my intern year, I have received sufficient direct observation and feedback on: **performing an informed consent** |  |  |  |  |  |
| Thus far during my intern year, I have received sufficient direct observation and feedback on: **creating and delivering signout** |  |  |  |  |  |
| The Intern Skills OSCE was a valuable learning exercise |  |  |  |  |  |
| The OSCE format made this a more valuable exercise than a classroom-based lecture |  |  |  |  |  |
| The Intern Skills OSCE should be continued for interns in future years |  |  |  |  |  |
| I feel confident in my ability to **create and deliver signout on my patients** |  |  |  |  |  |
| I feel confident in my ability to **call consults for my patients** |  |  |  |  |  |
| I feel confident in my ability to **triage and respond to pages from nursing staff** |  |  |  |  |  |
| I feel confident in my ability to do an **informed consent for a blood transfusion** |  |  |  |  |  |

Optional: Please share any other comments about today’s OSCE__________________________________
