# Supplementary material for: Development and Implementation of an OSCE for Formative Assessment of Core Clinical Skills in Internal Medicine Interns
Source: MedEdPORTAL. 2026 Feb 20;22:11576. doi: 10.15766/mep_2374-8265.11576 (PMC12920606; doi:10.15766/mep_2374-8265.11576)
Supplement: Supplementary file 1 — Prebrief Guide.docxStation A - GI Case Instructions.docxStation A - ID Case Instructions.docxStation A - GI Facilitator Guide.docxStation A - ID Facilitator Guide.docxStation B - Instructions.docxStation B - SP Case.docxStation B - SP Guide.docxStation C - Instructions.docxStation C - Sign-Out Template.docxStation C - Facilitator Guide.docxStation D - Instructions.docxStation D - Orders Form.docxStation D - Facilitator Guide.docxStation D - Page Delivery Instructions.docxStation A - Evaluator Checklist.docxStation B - Evaluator Checklist.docxStation C - Evaluator Checklist.docxStation D - Evaluator Checklist.docxPre- and Postsurveys.docx [file mep_2374-8265.11576-s001.zip › B. Station A - GI Case Instructions.docx]

**Appendix B: Station A – Calling a Consult**

**Intern Instructions**

You are the intern on the general medicine ward caring for patients. Your task at this station is to review the clinical note and call a medical subspecialist for a consult. **We will not use an initial consult page; you will just call the GI Fellow directly.** You should include relevant details as you would in a real-life scenario. Please use the phone in the room and place it on speaker; the call back number is written next to the phone.

You will have 15 minutes to review the clinical note, formulate your consult question, and call the consultant.

**GI Case Note**

**Case Summary:** 68 yo female (Tina Lee, MRN 3344775) with hx of COPD on baseline 2L O2, poorly controlled T2DM, chronic pain on opiates, and morbid obesity, who presented to OSH ED today with epigastric and RUQ pain and altered mental status. She was brought via EMS due to family concerns for drowsiness. In the ED was oriented to self, vomited x 1, and indicated she was in pain, otherwise history was difficult to obtain. Septic appearing with sinus tachy to 120s, BP 100/50, T 39.2, and O2 sat 85% on home 2L, improved to 90% on 4L. Labs demonstrated elevated LFTs and CT showed dilated CBD and enlarged gall bladder concerning for obstructing stone. Blood cultures ordered and pt was given fluids & Zosyn. Transferred to GMed service for escalation of cares and GI consultation.

**Your evaluation:** Tina reports ongoing RUQ pain and is confused, therefore history is difficult to obtain. You call her next-of-kin who confirms the above history and the medication list.

Home medications: Trelegy Ellipta inhaler, albuterol nebs PRN, metformin, insulin glargine 20 units bid, insulin lispro 10 units tid w/meals, morphine ER 30 mg bid, morphine IR 15 mg tid PRN.

Current vitals: HR 115, BP 100/50, T 38.5, RR 18, O2 91% on 4L, weight is 305 lb

Gen: Obese, fatigued appearing middle aged female in mild distress

HEENT: Scleral icterus present, pupils are pinpoint but reactive, mucous membranes are tacky

Pulm: Poor air movement bilaterally, no wheezing or rales

CV: Tachycardic, regular rhythm, no m/r/g

Abd: Soft, nondistended, TTP in the RUQ

Ext: No pitting edema in bilateral lower extremities, no joint swelling or erythema

Neuro: A&Ox2, does not know where she is currently but knows month & her name. Face symmetric, moving all extremities, gross sensation intact throughout.

Labs:

VBG: pH 7.38, PCO2 60 (baseline for pt)

Lactate: 3.5

Na 134, K 4.2, Cl 98, Bicarb 20, BUN 18, Cr 1.4 (prior was 1.3)

AST 200, ALT 250, T bili 6.5, Alk phos 250

WBC 14K (80% PMNs), Hgb 13, Plt 205

CT A/P outside read: CBD dilated to 3 cm with obstructing stone visualized in distal CBD. Dilated intrahepatic ducts. Gall bladder distended with stones present, no associated stranding or wall edema.

**Your senior instructs you to consult GI for this patient’s choledocholithiasis and ascending cholangitis.**
