# Supplementary material for: Development and Implementation of an OSCE for Formative Assessment of Core Clinical Skills in Internal Medicine Interns
Source: MedEdPORTAL. 2026 Feb 20;22:11576. doi: 10.15766/mep_2374-8265.11576 (PMC12920606; doi:10.15766/mep_2374-8265.11576)
Supplement: Supplementary file 1 — Prebrief Guide.docxStation A - GI Case Instructions.docxStation A - ID Case Instructions.docxStation A - GI Facilitator Guide.docxStation A - ID Facilitator Guide.docxStation B - Instructions.docxStation B - SP Case.docxStation B - SP Guide.docxStation C - Instructions.docxStation C - Sign-Out Template.docxStation C - Facilitator Guide.docxStation D - Instructions.docxStation D - Orders Form.docxStation D - Facilitator Guide.docxStation D - Page Delivery Instructions.docxStation A - Evaluator Checklist.docxStation B - Evaluator Checklist.docxStation C - Evaluator Checklist.docxStation D - Evaluator Checklist.docxPre- and Postsurveys.docx [file mep_2374-8265.11576-s001.zip › C. Station A - ID Case Instructions.docx]

**Appendix C: Station A – Calling a Consult**

**Intern Instructions**

You are the intern on the general medicine ward caring for patients. Your task at this station is to review the clinical note and call a medical subspecialist for a consult. **We will not use an initial consult page; you will just call the ID Fellow directly.** You should include relevant details as you would in a real-life scenario. Please use the phone in the room and place it on speaker; the call back number is written next to the phone.

You will have 15 minutes to review the clinical notes, formulate your consult question, and call the consultant.

**ID Case Note**

**Case summary**: Ted Franklin (MRN 7894992) is a 78-year-old man who was admitted to a local hospital in central Wisconsin four days ago with fever and altered mental status of 12 hours duration, brought into the ED via EMS when visited by his son and grandchildren. No known recent illnesses. In the emergency department locally he was disoriented and febrile, heart rate was 99 and BP was 108/60. He was saturating well on room air. Blood cultures were drawn and a non-contrasted CT head was obtained. He was started on vancomycin and cefepime in the ED.

By the next morning, his mental status had not improved and now his heart rate had risen to the 110s with systolic blood pressures in the 80s, requiring transfer to the local ICU and a call to the TLC for transfer.

On hospital day #3, he was transferred to the TLC. He required norepinephrine for about 8 hours but was weaned off yesterday evening.

Late yesterday, your team was called for a transfer of this patient to the IMC given acute need for ICU beds. When you assessed him early this morning, he was still altered and appeared to be grimacing in pain with any rolling in the bed or requests for volitional movement. He remains disoriented but easily arousable to voice and tactile stimulation. The TLC had empirically escalated his antibiotics from vancomycin and cefepime to vancomycin and meropenem.

**Medications and Allergies:**

Warfarin

Metformin

Atorvastatin

Lisinopril

No known drug allergies

**Past medical history:**

Aortic stenosis s/p TAVR 2019

Type 2 DM on metformin, complicated by mild peripheral neuropathy

CKD stage III

Atrial fibrillation

Hyperlipidemia

Hypertension

VS in IMC: Tmax last 24 hours 100.8F, HR 108, BP 100/70, RR 18, Saturating 91% RA

On exam, he is sleeping but arouses with voice and tactile stimulation; does not recall where he is or why he is in the hospital. Grimaces with movement about the bed. Audible systolic murmur heard throughout the precordium. TTP cervical spine. Irritable ROM left knee which is swollen and warm compared to the right. Grade III DFU right great toe.

Labs: Na 133, K 4.7, Cl 100, HCO3 22, BUN 40, Cr 2.3. WBC 11k, Hb 11.7, Plt 140. Lactate 2.4.

Blood cultures day of admission and day #2 positive for Staphylococcus aureus, no susceptibility testing has been faxed yet. Blood cultures from arrival yesterday NGTD.

Imaging: Normal CXR, Normal non-contrasted CT head.

**Progress Note, Hospital Day #4**

Interval history: Transferred out of TLC late yesterday; remains altered, only groans/grimaces in response to questions. Family at bedside, confirm normal mentation until ~12 hours preceding admission.

Medications:

Warfarin

Metformin (held)

Atorvastatin

Lisinopril (held)

Vancomycin (day 3)

Meropenem (day 3)

Exam:

VS: Tmax last 24 hours 100.8F, HR 108, BP 100/70, RR 18, Saturating 91% RA

Sleeping but arouses with voice and tactile stimulation. Grimaces with movement about the bed. Audible systolic murmur heard throughout the precordium. TTP cervical spine. Irritable ROM left knee which is swollen and warm compared to the right. Grade III DFU right great toe.

Labs: Na 133, K 4.7, Cl 100, HCO3 22, BUN 40, Cr 2.3. WBC 11k, Hb 11.7, Plt 140. Lactate 2.4.

Blood cultures here negative; reportedly MSSA from OSH ED

Imaging: Normal CXR, Normal non-contrasted CT head.

Assessment:

78 yo with history of AS s/p TAVR, HTN, HLD, a fib, CKD stage III and DM2 with last HbA1C 9.2% admitted with AMS and fever.

#AMS: Likely due to sepsis. CT head neg, urine cx neg.

- Monitor

#MSSA bacteremia: 2/2 blood cx + from OSH.

- Continue vanc/mero
- Call ID consult in AM
- Repeat blood cx here NGTD

#AKI: likely 2/2 sepsis

- Monitor
- Gentle hydration given AS

#Knee pain: gout v infections v arthritis

- Pain control
- Consider ortho v rheum consult

#Diabetic foot ulcer: Doesn’t appear acutely infected.

- Consider MRI
- Outpatient podiatry

Chronic medical conditions:

#Hypertension:

- Lisinopril held for AKI/hypotension

#Hyperlipidemia:

- Continue atorvastatin

#Aortic stenosis s/p TAVR:

- Continue warfarin

#Atrial fibrillation:

- Telemetry
- Continue warfarin

#Type 2 DM: Mild hyperglycemia in the hospital

- Insulin sliding scale
- Metformin held (AKI)

Diet: NPO given AMS

DVT ppx: Warfarin

Code status: Presumed full

**Your senior instructs you to consult ID for this patient for bacteremia.**
