# Supplementary material for: Development and Implementation of an OSCE for Formative Assessment of Core Clinical Skills in Internal Medicine Interns
Source: MedEdPORTAL. 2026 Feb 20;22:11576. doi: 10.15766/mep_2374-8265.11576 (PMC12920606; doi:10.15766/mep_2374-8265.11576)
Supplement: Supplementary file 1 — Prebrief Guide.docxStation A - GI Case Instructions.docxStation A - ID Case Instructions.docxStation A - GI Facilitator Guide.docxStation A - ID Facilitator Guide.docxStation B - Instructions.docxStation B - SP Case.docxStation B - SP Guide.docxStation C - Instructions.docxStation C - Sign-Out Template.docxStation C - Facilitator Guide.docxStation D - Instructions.docxStation D - Orders Form.docxStation D - Facilitator Guide.docxStation D - Page Delivery Instructions.docxStation A - Evaluator Checklist.docxStation B - Evaluator Checklist.docxStation C - Evaluator Checklist.docxStation D - Evaluator Checklist.docxPre- and Postsurveys.docx [file mep_2374-8265.11576-s001.zip › D. Station A - GI Facilitator Guide.docx]

**Appendix D: Station A – Calling a Consult**

**GI Fellow Facilitator Guide**

Our goals for the intern in this activity include:

1. Communicate clearly and concisely.
2. Relay all critical information regarding the consultation.
3. Recognize urgency/priority of patient situation.
4. Advocate for timely consultation.
5. Acknowledge and confirm preliminary recommendations.

While we can’t perfectly script this scenario, we encourage you to push learners to help achieve the goals above:

1. Ensure they’ve introduced themselves and their service.
2. Ask a few probing questions to get at urgency/acuity.
   1. For example:
      1. Vital signs
      2. Whether patient looks sick or not sick
      3. Imaging reports
      4. Does intern have awareness of pulmonary risk for anesthesia for this patient?
3. Provide pushback or education about when the ERCP is going to be done and whether the patient is appropriately stabilized (mostly gentle, use your judgment about how skilled or aware this intern seems).
   1. Consider:
      1. “This ERCP won’t be able to be performed at this time, what are you doing to stabilize the patient?”
      2. “Have you considered an alternate plan besides ERCP while this patient is unstable?” i.e. perc chole tube
4. Provide a long preliminary list of recommendations to prompt the intern to clarify/repeat back. Prompt them to discuss a follow-up plan.
   1. Examples: Clarify antibiotics, fluids, follow-up labs, place order for ERCP vs perc chole tube & IR consult (and whether that should happen overnight or the next day). Discuss need for OR vs ambulatory procedure center for ERCP based on stability.

After the conclusion of the consulting process, provide the intern feedback on the following:

1. Their initial contact – how they introduced themselves, whether their opening statement included the right information.
2. The patient presentation – was it concise but still included all relevant information?
3. Did they ask a clear question or request a procedure?
4. Was the discussion of the plan collaborative?
5. Did you feel they understood your recommendations and knew what to do?
