# Supplementary material for: Development and Implementation of an OSCE for Formative Assessment of Core Clinical Skills in Internal Medicine Interns
Source: MedEdPORTAL. 2026 Feb 20;22:11576. doi: 10.15766/mep_2374-8265.11576 (PMC12920606; doi:10.15766/mep_2374-8265.11576)
Supplement: Supplementary file 1 — Prebrief Guide.docxStation A - GI Case Instructions.docxStation A - ID Case Instructions.docxStation A - GI Facilitator Guide.docxStation A - ID Facilitator Guide.docxStation B - Instructions.docxStation B - SP Case.docxStation B - SP Guide.docxStation C - Instructions.docxStation C - Sign-Out Template.docxStation C - Facilitator Guide.docxStation D - Instructions.docxStation D - Orders Form.docxStation D - Facilitator Guide.docxStation D - Page Delivery Instructions.docxStation A - Evaluator Checklist.docxStation B - Evaluator Checklist.docxStation C - Evaluator Checklist.docxStation D - Evaluator Checklist.docxPre- and Postsurveys.docx [file mep_2374-8265.11576-s001.zip › E. Station A - ID Facilitator Guide.docx]

**Appendix E: Station A – Calling a Consult**

**ID Fellow Facilitator Guide**

Our goals for the interns in this activity are to:

1. Communicate clearly and concisely.
2. Relay all critical information regarding the consultation.
3. Recognize urgency/priority of patient situation.
4. Advocate for timely consultation.
5. Acknowledge and confirm preliminary recommendations.

While we can’t perfectly script this scenario, we encourage you to push learners to help achieve the goals above:

1. Ensure they’ve introduced themselves and their service.
2. Ask a few probing questions to get at urgency/acuity.
   1. For example:
      1. Vital signs
      2. Look sick/not sick
      3. Blood culture results
      4. Current anti-infectives
3. Provide pushback (mostly gentle, but use your judgment based on how skilled the intern seems) to provide them the opportunity to advocate for timely consultation.
   1. Consider:
      1. “We’ve already got 8 consults today, can I see this tomorrow?”
      2. “Sounds like they are on the right antibiotics, call me tomorrow if you still need me.”
4. Provide a long preliminary list of recommendations to prompt them to clarify/repeat back.
   1. Repeat blood cultures
   2. Change in antibiotics (tell them to stop vancomycin and meropenem and start oxacillin 2 g every 4 hours)
   3. MRI brain
   4. MRI of the right foot
   5. MRI cervical spine
   6. Transesophageal echocardiogram
   7. Orthopedics consultation
5. Prompt them to articulate a follow-up plan if not offered – who to call, by when, what to do if clinical status changes.

After the conclusion of the consulting process, provide the intern feedback on the following:

1. Their initial contact – how they introduced themselves, whether their opening statement included the right information.
2. The patient presentation – was it concise but still included all relevant information?
3. Did they ask a clear question or request a procedure?
4. Was the discussion of the plan collaborative?
5. Did you feel they understood your recommendations and knew what to do?
