# Supplementary material for: Development and Implementation of an OSCE for Formative Assessment of Core Clinical Skills in Internal Medicine Interns
Source: MedEdPORTAL. 2026 Feb 20;22:11576. doi: 10.15766/mep_2374-8265.11576 (PMC12920606; doi:10.15766/mep_2374-8265.11576)
Supplement: Supplementary file 1 — Prebrief Guide.docxStation A - GI Case Instructions.docxStation A - ID Case Instructions.docxStation A - GI Facilitator Guide.docxStation A - ID Facilitator Guide.docxStation B - Instructions.docxStation B - SP Case.docxStation B - SP Guide.docxStation C - Instructions.docxStation C - Sign-Out Template.docxStation C - Facilitator Guide.docxStation D - Instructions.docxStation D - Orders Form.docxStation D - Facilitator Guide.docxStation D - Page Delivery Instructions.docxStation A - Evaluator Checklist.docxStation B - Evaluator Checklist.docxStation C - Evaluator Checklist.docxStation D - Evaluator Checklist.docxPre- and Postsurveys.docx [file mep_2374-8265.11576-s001.zip › F. Station B - Instructions.docx]

**Appendix F: Station B – Informed Consent**

**Intern Instructions**

You are the night cross-cover intern at the VA.

You received the following sign-out from the VA Med Team 3:

| Summary | Day to Do | Night to Do |
| --- | --- | --- |
| Chris Anderson is a 64 yo with CAD, CKD3, afib on Eliquis, who presented with weakness and SOB. New anemia, Hgb 8.4 from 12. No symptoms of GIB as outpt. | NTD | [ ] Hgb at 21:00 |

You receive a page at 21:30: “Med3 pt BB, Hgb 6.4, transfuse? Jill, 75334”

You call back and talk with Jill – the pt is currently hemodynamically stable with normal vital signs, oriented, but she noted that the patient had one large melenic stool since admission. You realize no blood transfusion consent has been obtained. You order a type & screen and head to the patient’s room.

You will have 15 minutes to discuss with the patient and obtain consent for a blood transfusion.
