# Supplementary material for: Development and Implementation of an OSCE for Formative Assessment of Core Clinical Skills in Internal Medicine Interns
Source: MedEdPORTAL. 2026 Feb 20;22:11576. doi: 10.15766/mep_2374-8265.11576 (PMC12920606; doi:10.15766/mep_2374-8265.11576)
Supplement: Supplementary file 1 — Prebrief Guide.docxStation A - GI Case Instructions.docxStation A - ID Case Instructions.docxStation A - GI Facilitator Guide.docxStation A - ID Facilitator Guide.docxStation B - Instructions.docxStation B - SP Case.docxStation B - SP Guide.docxStation C - Instructions.docxStation C - Sign-Out Template.docxStation C - Facilitator Guide.docxStation D - Instructions.docxStation D - Orders Form.docxStation D - Facilitator Guide.docxStation D - Page Delivery Instructions.docxStation A - Evaluator Checklist.docxStation B - Evaluator Checklist.docxStation C - Evaluator Checklist.docxStation D - Evaluator Checklist.docxPre- and Postsurveys.docx [file mep_2374-8265.11576-s001.zip › G. Station B - SP Case.docx]

Appendix G: Standardized Patient Case Development Tool

**Date**: October 2, 2024

**Primary Case Author**: Samantha Murray-Bainer

**Secondary Case Author**: Alexandra Wick

**Standardized Patient Educator**: Jenna Patenaude

**Name of Case**: Symptomatic anemia due to GI bleed in need of a blood transfusion

**Name of Educational and/or Assessment Activity:** Development and Implementation of an OSCE for Formative Assessment of Core Clinical Skills in Internal Medicine Interns

**Patient Name:** Chris Anderson

**Chief Complaint**: Weakness and shortness of breath

**Most Likely Diagnosis and Differential With Rationale From History and/or Physical Exam:** Symptomatic anemia due to GI bleed

**Challenge Question:** Obtain informed consent for a blood transfusion

**Domains**:

- Professionalism

X Communication and Interpersonal Skills

- Medical History
- Physical Exam

X Shared Decision-Making

X Patient Education

- Clinical Reasoning
- Documentation
- Handoff
- Presentation
- Other:

**Type and Level of Learner:** Internal medicine interns

**Case Objectives:**

**By the end of this activity, learners will be able to:**

1. Describe the benefits and risks of a blood transfusion

2. List alternatives to blood transfusion

3. Engage in shared decision-making with a patient, considering their values and preferences

| **SETTING**: | Inpatient room |
| --- | --- |
| **PATIENT PROFILE:** | |
| Age range | 60-70 years |
| Religious/spiritual background | All may be used |
| Sex | Male/All may be used |
| Sexual orientation | All may be used |
| Gender expression | All may be used |
| Race and ethnicity | All may be used |
| Physical description | All may be used |
| Physical limitations | All may be used |
| Patient appearance | Dressed in hospital gown |
| Moulage + location | None |
| Affect | Pleasant and cooperative |
| Family group | All may be used |
| Education | All may be used |
| Level of health literacy | All may be used |
| Employment, if any | All may be used |
| Home/homeless | All may be used |
| Financial situation - any current stresses | All may be used |
| Insurance status | Veterans Affairs Health Care |
| Habits | All may be used |
| Activities | All may be used |
| Typical day - what is the usual daily routine | All may be used |

| **CASE INFORMATION** | |
| --- | --- |
| Chief Concern: What the patient will say when greeted by the student. The patient’s primary reason for seeking medical care often stated in their own words. | “I feel weak and short of breath.” |
| Additional Concerns: Other, if any, concerns the patient has today (i.e., symptoms, requests, expectations, etc.) that will become part of set agenda. | None |
| THE PATIENT’S STORY: The SP will be asked to tell their symptom story and the personal and emotion impact for each of their concerns. You will want to write this in the patient’s voice. The symptom story should be able to answer this question: “Tell me more about [chief concern/additional concern], starting at the beginning and bringing me up to now.”  The personal context should be able to answer questions concerning the broader personal/psychosocial context of symptoms, especially the patient’s beliefs/attributions.  The emotional context should be able to ask how are you doing with this, how does this make you feel, how has this affected you emotionally? IMPACT: How has this affected your life? How has this been for your family? | I am a 64-year-old veteran with worsening weakness and shortness of breath for the past few days. I came to the emergency room earlier today, and they found that I had low blood counts. They recommended I be hospitalized for further monitoring. The doctors don’t know why my blood counts are low. I have never had a blood transfusion before. A couple of hours ago I had a black, tarry stool that the nurses seemed worried about. |
| **HISTORY OF PRESENT ILLNESS:** | |
| Onset (when; gradual or sudden) | Gradual increase in weakness and shortness of breath over the last few days  Black stool suddenly a couple of hours ago |
| Setting (what was going on or where was patient when symptoms first noticed?) | All may be used |
| Duration (how long) | Couple of days for weakness and shortness of breath  One episode of black stool a couple of hours ago |
| Time relationships (frequency, constant or intermittent) | Constant feeling of weakness and shortness of breath |
| Location | Generalized weakness, feels short of breath in the lungs |
| Radiation | N/A |
| Quality | Feels like they can’t get air fully into their lungs with breaths  One black, tarry stool |
| Amount | “It seemed like a lot, but I’m not sure how to know how much.” |
| Aggravated by what | Going up stairs, walking quickly |
| Relieved by what | None |
| Associated with what | None |
| Attitude (what does the patient think is the problem, and how do they feel about it) | Pleasant and cooperative, inquisitive |
| Overall course | Gradually worsening symptoms over the last few days, prompting presentation |
| **REVIEW OF SYSTEMS:** Significant positives and negatives | |
| General | + fatigue and generalized weakness, no fevers or chills |
| Cardiovascular | no chest pain or pressure, palpitations, shortness of breath when lying down, no leg swelling |
| Respiratory | +shortness of breath and dyspnea on exertion, no cough or wheezing |
| Musculoskeletal | No joint pain or swelling |
| Neurologic | No numbness or tingling |
| Gastrointestinal | +black, tarry stool, no bright red blood in the stool, abdominal pain, nausea, or vomiting |
| Past medical history | Coronary artery disease with heart stent placed 5 years ago  Chronic kidney disease stage 3  Atrial fibrillation (abnormal heart rhythm) |
| Medication allergies (name and reaction) | None |
| Environmental allergies (name and reaction) | None |
| Illnesses | None |
| Vaccinations | All may be used |
| Surgeries | None |
| Accidents/injuries/trauma | None |
| Hospitalization | None |
|  | |
| Inclusive sexual and reproductive history | |
| Sexual practices  Sexual partners  Protection: Use of safer sex practices  Use of birth control if appropriate  Risk of intimate partner violence | N/A |
| OB/GYN history | N/A |
| Medications | Apixaban 5 mg twice daily for atrial fibrillation  Aspirin 81 mg daily |
| Immunizations | All may be used |
| Tobacco products:   - Cigarettes - Cigar - Pipe - Chew - E-cigarettes | X Never   - Past - year started/year quit - Current   - Quantity   - # of years |
| Alcohol   - Beer - Wine - Liquor - Other | X Never   - Past - year started/year quit - Current   - Quantity   - # of years |
| Drugs   - Weed - Cocaine - Heroin - Meth - IV - Inhalants - Other | X Never   - Past - year started/year quit - Current   - Quantity   - # of years |
| Diet (describe) | All may be used |
| Exercise (describe) | All may be used |
| List any other important social history or information important to this case | None |
| Family history |  |
| Mother, father, siblings, grandparents, and other significant findings | Mother had an allergic reaction to a blood transfusion |
|  |  |
| Physical Exam  The SP should sit comfortably in the bed. The intern should not perform an exam as it is not an expectation for this station. | |
| **PHYSICAL EXAM FINDINGS** |  |
| 1. Written in layperson’s terms | Patient is lying comfortably in bed, can sit up to talk with intern |
| 1. General appearance - affect, appearance, position of patient at opening (i.e., sitting, lying down, holding abdomen, etc.) | Patient is alert and cooperative, breathing comfortably on room air |
| 1. Vital signs | N/A |
| 1. Specific findings and affect | N/A |
| 1. Response to certain physical movements | N/A |
|  |  |
| **DIAGNOSIS AND DIFFERENTIAL** |  |
| Diagnosis with support from positive and negative history and PE findings | Symptomatic anemia due to GI bleed, likely due to an upper source due to melena |
| Differential with support from positive and negative history and PE findings | N/A |
|  |  |
| **MANAGEMENT OR DIAGNOSTIC PLAN** | Intern will recommend a blood transfusion. They will provide alternatives to blood transfusion including continuing to monitor blood counts and symptoms, giving fluids, and giving IV iron. |
|  |  |
| **PROFESSIONALISM ISSUES OR CHALLENGES** | Patient should question who the intern is because this will be the first time meeting each other, despite the patient meeting other doctors earlier in the day. Patient should ask several questions as listed in SP instructions appendix. |
