# Supplementary material for: Development and Implementation of an OSCE for Formative Assessment of Core Clinical Skills in Internal Medicine Interns
Source: MedEdPORTAL. 2026 Feb 20;22:11576. doi: 10.15766/mep_2374-8265.11576 (PMC12920606; doi:10.15766/mep_2374-8265.11576)
Supplement: Supplementary file 1 — Prebrief Guide.docxStation A - GI Case Instructions.docxStation A - ID Case Instructions.docxStation A - GI Facilitator Guide.docxStation A - ID Facilitator Guide.docxStation B - Instructions.docxStation B - SP Case.docxStation B - SP Guide.docxStation C - Instructions.docxStation C - Sign-Out Template.docxStation C - Facilitator Guide.docxStation D - Instructions.docxStation D - Orders Form.docxStation D - Facilitator Guide.docxStation D - Page Delivery Instructions.docxStation A - Evaluator Checklist.docxStation B - Evaluator Checklist.docxStation C - Evaluator Checklist.docxStation D - Evaluator Checklist.docxPre- and Postsurveys.docx [file mep_2374-8265.11576-s001.zip › H. Station B - SP Guide.docx]

**Appendix H: Station B – Informed Consent**

**Standardized Patient Instructions**

**Case summary:** You are an elderly patient (Chris Anderson) with heart disease, kidney disease, and atrial fibrillation (abnormal heart rhythm) and take multiple medications that make you more likely to bleed. You came to the VA Emergency Room with a few days of worsening weakness and shortness of breath and were found to be anemic with lower blood count than your baseline. You were admitted to the hospital earlier today. It’s now later in the evening and the admitting medical team ordered a repeat lab draw to check your blood count to make sure it isn’t dropping further. It has dropped enough that a blood transfusion is recommended. Because it is now after hours, your main medical team is no longer there for the day, and the intern physician on night cross-cover is going to talk to you about a blood transfusion and should ask you to sign a consent form. You have never had a blood transfusion before, and all you know about them is that people could get infections like HIV from blood.

**You may ad lib questions during any of these portions, examples provided below.**

1. Intern should introduce themselves and why they are coming to see you
   1. Example Question: “I don’t remember you from the ER, are you one of my doctors?”
2. Intern should break the news you are becoming significantly anemic and that you would benefit from a blood transfusion.
   1. Example Questions: “Where is the blood loss coming from?” “How long does it take to get a blood transfusion?”
3. The intern should explain the following **risks** of blood transfusion (in any order):
4. Allergic reaction to blood products: Likelihood: most likely reaction to occur to blood. Prevention: testing your blood (something called a type & screen) to prevent major reactions, and also RN monitors while giving blood to check for reactions like fever or shortness of breath.
   1. Example Questions: “My mother had an allergic reaction to a blood transfusion, will that happen to me?” “Can anything be done to prevent an allergic reaction?”
5. Transmission of infections like HIV and Hepatitis**:** Likelihood: unlikely, <1:1,000,000 these days. Prevention: all blood products are screened routinely for infections
   1. Example Questions: “Can I contract Lyme disease from it?” “Can I contract COVID from it?”
6. They *may* bring up additional reactions like volume overload, routine fever reaction.
7. The interns should explain the **benefits** of blood transfusion:
8. Improve how you are feeling in terms of your shortness of breath and weakness
9. Something along the lines of protecting your organs or delivering enough oxygen to your body.
10. Ideally, the intern should bring up **alternatives (examples below):** No transfusion and continuing to check your blood counts and monitor for worsening symptoms. Alternatives: fluids and IV iron. These are not ideal when you are severely anemic from blood loss that is actively occurring.
    1. **If they do not, ask: “Can something else be done instead of blood?”**
11. Finally, the intern should **check for your understanding, allow for questions, and have you sign the consent form.**
    1. Ad lib any questions you may have.
