# Supplementary material for: Development and Implementation of an OSCE for Formative Assessment of Core Clinical Skills in Internal Medicine Interns
Source: MedEdPORTAL. 2026 Feb 20;22:11576. doi: 10.15766/mep_2374-8265.11576 (PMC12920606; doi:10.15766/mep_2374-8265.11576)
Supplement: Supplementary file 1 — Prebrief Guide.docxStation A - GI Case Instructions.docxStation A - ID Case Instructions.docxStation A - GI Facilitator Guide.docxStation A - ID Facilitator Guide.docxStation B - Instructions.docxStation B - SP Case.docxStation B - SP Guide.docxStation C - Instructions.docxStation C - Sign-Out Template.docxStation C - Facilitator Guide.docxStation D - Instructions.docxStation D - Orders Form.docxStation D - Facilitator Guide.docxStation D - Page Delivery Instructions.docxStation A - Evaluator Checklist.docxStation B - Evaluator Checklist.docxStation C - Evaluator Checklist.docxStation D - Evaluator Checklist.docxPre- and Postsurveys.docx [file mep_2374-8265.11576-s001.zip › I. Station C - Instructions.docx]

**Appendix I: Station C – Sign-Out**

**Intern Instructions**

After a busy day of discharging, you are currently caring for 2 patients and getting ready to sign out for the day. You will have 10 minutes to review Case 1 and Case 2 and create a written sign-out. The senior resident will then enter, and you will have 5 minutes to provide a verbal sign-out to the resident who will be covering overnight using the I-PASS method.

Please write your written sign-out on the document uploaded to the desktop. Leave this document open on your screen at the end of the session.

**Case 1 Progress Note – D Reyes**

**Subjective:**

This morning, breathing is improving significantly. No chest pain, cough has resolved. Feeling very shaky, irritable and sweaty. Notes she saw a dog in her room that she knows isn’t there. Has been hearing conversations overnight. CIWAs over past 24 hours range from 10-24, required lorazepam x 6.

**Exam:**

VS Tmax 37.8, HR 95, BP 156/70, RR 24, sat 95% on 4L NC

GEN: Appears uncomfortable, sweaty, sitting up in chair

HEENT: normocephalic/atraumatic, no scleral icterus

RESP: Rhonchi at R base, improving; no wheezes, non-labored on 4L NC

CV: Irregular rhythm, normal rate, no murmurs, rubs or gallops, 1+ LE edema bilaterally, JVP mildly elevated

GI: Soft, non-tender, non-distended, bowel sounds present

EXT: Warm, well-perfused, moves all extremities

NEURO: Tremors of bilateral upper extremities, moves all extremities equally, awake, alert, oriented x 4

PSYCH: + auditory and visual hallucinations

DERM: Warm, diaphoretic

Labs:

WBC 7.5 from 15 on admission

Hgb 13.2- stable

Cr 0.6 from 1.1 on admission

Blood cultures and sputum cultures remain no growth to date

**Assessment/Plan**

D Reyes is a 54-year-old with a past medical history of COPD on 2L NC at rest, HFpEF (EF 60%), DM II, A fib on xarelto, HTN, EtOH use disorder who presented 2 days ago with dyspnea, found to have acute hypoxic respiratory failure 2/2 community acquired pneumonia, a fib with RVR and AKI, now improving with IV antibiotics and fluids. Course has been complicated by EtOH withdrawal.

**#Acute hypoxic respiratory failure 2/2 Community acquired pneumonia:** CXR w/ RLL infiltrate. White count down-trending. Oxygen weaned from HFNC now down to 4L

- Continue Ceftriaxone and azithromycin

- Wean oxygen with goal sats of 88%+

- RT per protocol

- Follow-up sputum and blood cultures (no growth to date)

**#EtOH use disorder c/b withdrawal:** Developing worsening withdrawal symptoms. Prior hx of withdrawal seizures as well as an ICU stay for DTs in 2020. Last EtOH use shortly prior to admission.

- Continue CIWA with lorazepam PRN

**#AKI:** Likely prerenal, improving after careful fluid resuscitation.

- Hold lasix and losartan

- Trend creatinine daily

- Avoid nephrotoxic agents

**#Atrial fibrillation w/ RVR, now rate controlled:** RVR on admission, now rate controlled after IV fluids and treatment of PNA.

- Continue home diltiazem

- Continue home rivaroxaban

**Chronic medical conditions:**

**#HFpEF:** EF 60% 3 months ago. SGLT2 cost prohibitive. Starting to develop lower extremity edema after IV fluids for PNA.

- Holding lasix due to AKI

**#DM II**

- Continue lantus 30 units daily, SSI TID AC and HS, ICR 1:30 with meals

**#COPD:** Not in acute exacerbation

- Continue home inhalers

**#HTN:**

- Continue diltiazem

- Holding losartan and lasix due to AKI

FEN: Salt controlled, diabetic diet

VTE ppx: Therapeutic anticoagulation above

Access: PIV

Dispo: Home with home health PT/OT pending improvement in respiratory status

Code status: FULL, discussed with patient on admission

**Case 2 H&P - S Baylor**

**CC**: Altered mental status, fever

**HPI:**

Ms. Baylor is a 95-year-old female with a history of dementia with activated HCPOA, A fib on Xarelto, recurrent MDR UTIs, HTN, and DM II who presents from her nursing facility with increased somnolence and AMS x 2 days as well as fever

History obtained from chart and discussion with memory care facility staff as patient unable to provide history.

Ms Baylor was in her usual state of health until 3 days ago when staff at the facility began to notice she was sleeping all day and skipping meals. She became increasingly lethargic and stopped drinking or taking her medications yesterday. She was not urinating as frequently. This morning, she was noted to be warm and her temperature was checked and was 38.6. Her facility called EMS who noted she was hypoxic on room air to 82% and brought her to the ED. Her POC glucose was 78.

**ROS**

ROS was unable to be obtained 2/2 patient mental status

**Past Medical History:**

Dementia with activated HCPOA

Atrial fibrillation on apixaban

HTN

DM II

Osteoarthritis

Recurrent UTIs

Urge incontinence

Breast cancer in 1995 s/p bilateral mastectomies

**Past Surgical History:**

Cholecystectomy

Bilateral mastectomies

**Family History**

Unable to obtain

**Social History**

Lives in memory care facility. At baseline requires assistance with all ADLs and iADLs. Typically only oriented to self but enjoys talking with staff and visitors. Previously worked as a high school teacher. Per chart, no history of tobacco use in past.

**Meds:**

Donepezil 10 mg daily

Metoprolol succinate 25 mg daily

Apixaban 2.5 mg twice daily

Melatonin 10 mg daily

Metformin ER 500 mg daily

Acetaminophen 650 mg three times daily PRN pain

**Allergies:**

Penicillin- Anaphylaxis

**Exam**

**VS Tmax 38.4, HR 102, BP 100/60, RR 28, Sat 94% on 3L NC**

GEN: Somnolent, difficult to arouse, lying in bed

RESP: Rhonchi in R base, no wheezes or crackles, no accessory muscle usage on 3L NC

CV: irregular rhythm, tachycardic, no murmurs, rubs or gallops, no LE edema

GI: Soft, no obvious tenderness on palpation, non-distended, bowel sounds present

EXT: Warm, well-perfused

NEURO: Somnolent, grimaces and grunts, some spontaneous movement of UE

DERM: Warm, pale

**Labs**

WBC 17.3, Hgb 12.5, Plt 95

Na 137, K 3.8, HCO3 19, BUN 36, Cr 1.45 (baseline 1.2), glu 70

Lactate 2.5🡪1.8🡪1.5

CXR

Right lower lobe infiltrate on CXR

**Assessment/Plan**

S Baylor is a 95-year-old female with a history of dementia with activated HCPOA, A fib on Xarelto, recurrent MDR UTIs, HTN, and DM II who presents from her nursing facility with increased somnolence and AMS x 2 days, found to have sepsis and acute hypoxic respiratory failure secondary to RLL pneumonia

#**Sepsis and acute hypoxic respiratory failure 2/2 RLL pneumonia:** P/w leukocytosis, altered mental status, hypoxia requiring 3L and CXR w/ RLL infiltrate consistent with sepsis secondary to pneumonia. Prior hx of Pseudomonas PNA 1 year ago resistant to fluoroquinolones. s/p 2L IVF in ED, lactate down-trended.

- Continue ceftriaxone; low threshold to broaden to cefepime if clinical status worsens for pseudomonas coverage

- Follow-up sputum and blood cultures

- NPO pending improvement in mental status (failed bedside swallow in ED)

**#Toxic metabolic encephalopathy:** Likely 2/2 infection. Has not been taking meds for 2 days so unlikely to be medication induced.

- Supportive care

**#Atrial fibrillation:** Currently unable to take PO meds. Last TTE was 5 years ago with EF of 65% and no valvular disease.

- Hold metop until able to take PO

- For sustained HR >130, will give IV metoprolol

- Apixaban on hold until able to take PO meds

**#DM II**

- Hold home metformin while not taking PO

- Monitor glucoses, consider SSI if elevated

**#HTN**

- Hold home antihypertensives i/s/o sepsis

Code Status: DNR/ok to intubate, discussed with HCPOA on admission
