# Supplementary material for: Development and Implementation of an OSCE for Formative Assessment of Core Clinical Skills in Internal Medicine Interns
Source: MedEdPORTAL. 2026 Feb 20;22:11576. doi: 10.15766/mep_2374-8265.11576 (PMC12920606; doi:10.15766/mep_2374-8265.11576)
Supplement: Supplementary file 1 — Prebrief Guide.docxStation A - GI Case Instructions.docxStation A - ID Case Instructions.docxStation A - GI Facilitator Guide.docxStation A - ID Facilitator Guide.docxStation B - Instructions.docxStation B - SP Case.docxStation B - SP Guide.docxStation C - Instructions.docxStation C - Sign-Out Template.docxStation C - Facilitator Guide.docxStation D - Instructions.docxStation D - Orders Form.docxStation D - Facilitator Guide.docxStation D - Page Delivery Instructions.docxStation A - Evaluator Checklist.docxStation B - Evaluator Checklist.docxStation C - Evaluator Checklist.docxStation D - Evaluator Checklist.docxPre- and Postsurveys.docx [file mep_2374-8265.11576-s001.zip › J. Station C - Sign-Out Template.docx]

**Appendix J: Station C Written Sign-Out Template**

**Name___________________________**

**Instructions:** Using the information provided in the two case notes, create a written sign-out below for 2 patients. You will then provide a verbal sign-out to the resident who will be covering overnight using the I-PASS method.

| **Patient** | **Code Status** | **Handoff Summary** | **Handoff Action List/Sit Awareness/Contingency** | **Handoff Overnight** |
| --- | --- | --- | --- | --- |
| Reyes  #####  Room #30 |  | 54-year-old female with PMH of XXXX  02 needs/settings:  Drips:  Abx: | XC:  FYI:  Team |  |
| Baylor #####  Room #24 |  | 95-year-old female with PMH of XXXX  02 needs/settings:  Drips:  Abx: | XC:  FYI:  Team |  |
