# Supplementary material for: Development and Implementation of an OSCE for Formative Assessment of Core Clinical Skills in Internal Medicine Interns
Source: MedEdPORTAL. 2026 Feb 20;22:11576. doi: 10.15766/mep_2374-8265.11576 (PMC12920606; doi:10.15766/mep_2374-8265.11576)
Supplement: Supplementary file 1 — Prebrief Guide.docxStation A - GI Case Instructions.docxStation A - ID Case Instructions.docxStation A - GI Facilitator Guide.docxStation A - ID Facilitator Guide.docxStation B - Instructions.docxStation B - SP Case.docxStation B - SP Guide.docxStation C - Instructions.docxStation C - Sign-Out Template.docxStation C - Facilitator Guide.docxStation D - Instructions.docxStation D - Orders Form.docxStation D - Facilitator Guide.docxStation D - Page Delivery Instructions.docxStation A - Evaluator Checklist.docxStation B - Evaluator Checklist.docxStation C - Evaluator Checklist.docxStation D - Evaluator Checklist.docxPre- and Postsurveys.docx [file mep_2374-8265.11576-s001.zip › K. Station C - Facilitator Guide.docx]

**Appendix K: Station C – Sign-Out**

**Senior Resident Facilitator Guide**

You are receiving verbal sign-out from an intern on 2 patients you will be cross-covering overnight. They will have 10 minutes to read the cases and write sign-out. Then, you will enter the room, and they will have 5 minutes to deliver verbal sign-out to you.

Allow them to provide their verbal sign-out and ask clarifying questions at the end of the sign-out or earlier if you are asked/invited to do so by the intern. At the end of the sign-out, you and the faculty observer will provide feedback on areas that were performed well and 1-2 areas for improvement (5 minutes total for feedback).

Some potential areas to consider when providing feedback (you do not have to cover all of these):

1. Did you receive a tailored summary—e.g., the big picture of what you will need to prioritize overnight?
2. Did the handoff include:
   1. Identification as stable, watcher, or unstable
   2. Summary statement, hospital course, relevant elements of assessment and plan
   3. Action list- to-do list, timing, and who should perform each task
   4. Situational awareness or contingency planning- what might happen and what to do
   5. Ensuring your understanding, allowing for questions
3. How was the pace of the handoff? Too fast? Too Slow? Just right?
4. Was the level of detail appropriate? Too much information? Not enough information? – consider what information you would need to appropriately cross-cover this patient
