# Supplementary material for: Development and Implementation of an OSCE for Formative Assessment of Core Clinical Skills in Internal Medicine Interns
Source: MedEdPORTAL. 2026 Feb 20;22:11576. doi: 10.15766/mep_2374-8265.11576 (PMC12920606; doi:10.15766/mep_2374-8265.11576)
Supplement: Supplementary file 1 — Prebrief Guide.docxStation A - GI Case Instructions.docxStation A - ID Case Instructions.docxStation A - GI Facilitator Guide.docxStation A - ID Facilitator Guide.docxStation B - Instructions.docxStation B - SP Case.docxStation B - SP Guide.docxStation C - Instructions.docxStation C - Sign-Out Template.docxStation C - Facilitator Guide.docxStation D - Instructions.docxStation D - Orders Form.docxStation D - Facilitator Guide.docxStation D - Page Delivery Instructions.docxStation A - Evaluator Checklist.docxStation B - Evaluator Checklist.docxStation C - Evaluator Checklist.docxStation D - Evaluator Checklist.docxPre- and Postsurveys.docx [file mep_2374-8265.11576-s001.zip › L. Station D - Instructions.docx]

**Appendix L: Station D – Paging**

**Intern Instructions**

You are on cross-cover overnight on the general medicine ward and are responsible for multiple patients. The day team signed their patients out to you, and the sign-out document is available on the desktop for your reference. The EMR is on downtime so you must write orders on the paper form. You will receive multiple pages on the pager provided. Please call back the nurses using the phone in the room on speaker. The call-back number is located next to the phone and is the same for all pages.

Call back the highest-priority pages first and address the nurses’ concerns. You may not have time to call back every page.

- If you feel a bedside evaluation is necessary, let the nurse know you will go to the bedside in 15 minutes.
- If you feel that a callback is not necessary and that an order will suffice, please write your orders on the provided paper form.

This station will last 15 minutes.

| **Patient** | **Code Status** | **Handoff Summary** | **Handoff Action List/Sit Awareness/Contingency** | **Handoff Overnight** |
| --- | --- | --- | --- | --- |
| Kris Wallace  3436112  F6/562 | FULL | 68-year-old male with PMH of CAD s/p CABG (2017), HTN, HLD, tobacco use, and COPD who presents with shortness of breath found to have COPD exacerbation.  02 needs/settings: 2L NC  Drips: none  Abx: azithromycin | XC: NTD  FYI: Has had chest pain, thought to be due to pleuritis and coughing.  Team  [ ] ambulatory oximetry test before discharge |  |
| Elaine Bolt 7261160  B6/412 | DNR/  May Intubate | 89-year-old female with PMH of dementia (active HCA), hypothyroidism, depression, and CKD3 who presents with fall and non-operative pelvic fractures, AKI, and anemia.  02 needs/settings: 1L NC  Drips: NS at 75 ml/hr  Abx: none | XC: NTD  FYI: received 1u pRBC on admission, hgb stable since  Team  [ ] update HCA (daughter) daily |  |
| Ava Smith 3987992  F4/424 | FULL | 32-year-old female with PMH of obesity, DM2 on insulin, and Crohn’s disease on infliximab who presents with cellulitis of the left hand.  02 needs/settings: none  Drips: none  Abx: vancomycin, zosyn | XC:  [ ] f/u MRSA swab, if negative can discontinue vancomycin  FYI: IF called about pain, ok to give additional oxycodone.  Team  [ ] hand surgery recommendations |  |
| Henry Gates 1197524  B6/638 | FULL | 59-year-old male with PMH of HFpEF (EF 55%), AS s/p TAVR, HTN, HLD, pAF on apixaban, and DM2 who presents with CHF exacerbation undergoing diuresis.  02 needs/settings: 4L OxyMask  Drips: none  Abx: none | XC:  [ ] replete electrolytes  FYI: IF worsening hypoxemia or shortness of breath, can give additional 40 mg IV Lasix.  Team  [ ] f/u TTE |  |
| Riley Park 7800999  F6/578 | FULL | 25-year-old male with PMH of anxiety and alcohol use disorder who presents with abdominal pain due to pancreatitis.  02 needs/settings: none  Drips: LR at 150 ml/hr  Abx: none | XC: NTD  FYI: Peri-pancreatic fluid collection on imaging.  IF fevers, repeat infectious workup.  Stopped CIWA due to no evidence of alcohol withdrawal.  Team  [ ] advance diet |  |
