# Supplementary material for: Development and Implementation of an OSCE for Formative Assessment of Core Clinical Skills in Internal Medicine Interns
Source: MedEdPORTAL. 2026 Feb 20;22:11576. doi: 10.15766/mep_2374-8265.11576 (PMC12920606; doi:10.15766/mep_2374-8265.11576)
Supplement: Supplementary file 1 — Prebrief Guide.docxStation A - GI Case Instructions.docxStation A - ID Case Instructions.docxStation A - GI Facilitator Guide.docxStation A - ID Facilitator Guide.docxStation B - Instructions.docxStation B - SP Case.docxStation B - SP Guide.docxStation C - Instructions.docxStation C - Sign-Out Template.docxStation C - Facilitator Guide.docxStation D - Instructions.docxStation D - Orders Form.docxStation D - Facilitator Guide.docxStation D - Page Delivery Instructions.docxStation A - Evaluator Checklist.docxStation B - Evaluator Checklist.docxStation C - Evaluator Checklist.docxStation D - Evaluator Checklist.docxPre- and Postsurveys.docx [file mep_2374-8265.11576-s001.zip › M. Station D - Orders Form.docx]

**Appendix M: Station D Orders Form**

**Name**____________________________________

**Instructions:** If you decide not to call back the nurse and instead write orders to address the page, please write the orders below.

| **Name** | **Room** | **Orders** |
| --- | --- | --- |
| Kris Wallace | F6/562 |  |
| Elaine Bolt | B6/412 |  |
| Ava Smith | F4/424 |  |
| Henry Gates | B6/638 |  |
| Riley Park | F6/578 |  |
