# Supplementary material for: Development and Implementation of an OSCE for Formative Assessment of Core Clinical Skills in Internal Medicine Interns
Source: MedEdPORTAL. 2026 Feb 20;22:11576. doi: 10.15766/mep_2374-8265.11576 (PMC12920606; doi:10.15766/mep_2374-8265.11576)
Supplement: Supplementary file 1 — Prebrief Guide.docxStation A - GI Case Instructions.docxStation A - ID Case Instructions.docxStation A - GI Facilitator Guide.docxStation A - ID Facilitator Guide.docxStation B - Instructions.docxStation B - SP Case.docxStation B - SP Guide.docxStation C - Instructions.docxStation C - Sign-Out Template.docxStation C - Facilitator Guide.docxStation D - Instructions.docxStation D - Orders Form.docxStation D - Facilitator Guide.docxStation D - Page Delivery Instructions.docxStation A - Evaluator Checklist.docxStation B - Evaluator Checklist.docxStation C - Evaluator Checklist.docxStation D - Evaluator Checklist.docxPre- and Postsurveys.docx [file mep_2374-8265.11576-s001.zip › N. Station D - Facilitator Guide.docx]

**Appendix N: Station D – Paging**

**Nurse Facilitator Guide**

In this station, you will play the role of multiple nurses overnight on a general medicine floor. The intern will receive two batches of pages: pages 1-3 will be sent at the beginning of the station, and pages 4-5 will be sent after 8 minutes have elapsed. They will triage the pages and call you back using the phone in the room. **Please initially answer as the “unit front desk” and then locate the script** **associated with the nurse's name** they are requesting to speak with (see next page).

This station will last 15 minutes, followed by 5 minutes for feedback. Please go to the station room to provide feedback in person. Some potential areas to consider when providing feedback (you do not have to cover all these areas):

1. Did you feel that your concerns were heard?
2. Did the intern use open-ended questions?
3. Did the intern use closed-loop communication?
4. Did the intern demonstrate an appropriate balance of being inquisitive and decisive during the discussion?
5. Did you feel like an appropriate clinical decision was made?
6. If the intern did not know what to do, did they offer an acceptable next step (e.g., calling their senior/consultant, stating plan to see the patient to determine what to do next)?

**Page 1 – Jen RN**

**Page**: Wallace F6/562. I just noticed this patient hasn’t had a bowel movement in 3 days. Can we get a PRN? Jen.

**When intern calls, answer phone with**: “Unit front desk, how can I help you?” Then answer the phone as Jen.

**When intern specifies why they are calling, state:** “I was just looking through the chart and saw Mr. Wallace hasn’t had a bowel movement for 3 days. Can we get something for a bowel regimen?”

**If asked about specifically, the following should be provided:** If student asks for information you don’t have, state “I do not have that information.”

| One-Liner | 68M with history of CAD s/p CABG (2017), HTN, HLD, tobacco use, and COPD who was admitted 3 days ago with shortness of breath found to have COPD exacerbation. |
| --- | --- |
| Vitals | T 98.6 F  BP 138/86  HR 82  RR 16  SpO2 94% on 2L NC |
| Abdominal pain? | No |
| Nausea? | No |
| What medications is he on? | Prednisone, azithromycin, albuterol, duonebs |
| Assessment/How does he look? | Comfortable. Alert and oriented. No complaints. No abdominal pain. Breathing comfortably. |

**If intern states plan to order bowel regimen, reply:** “Thank you!”

**Page 2 – Sara RN**

**Page:** Bolt B6/412. Patient with increasing agitation, pulled out IV. Can you order something? Please call Sara.

**When intern calls, answer phone with**: “Unit front desk, how can I help you?” Then answer the phone as Sara.

**When intern specifies why they are calling, state:** “This patient is getting more and more agitated throughout the night. She pulled out her IV. Can we get some lorazepam so she can just sleep?”

Express frustration. You have already tried multiple types of redirection without success. The underlying cause of your frustration is concern that the patient may hurt herself, you, or other staff.

**If asked about specifically, the following should be provided.** If student asks for information you don’t have, state “I do not have that information.”

| One-Liner | 89F with dementia (active HCA), hypothyroidism, depression, and CKD3 who was admitted 2 days ago with fall and non-operative pelvic fractures, AKI, and anemia. |
| --- | --- |
| Vitals | T 98.2 F  BP 172/98  HR 99  RR 18  SpO2 96% on 1L NC |
| Access | 1 PIV |
| Assessment/How does patient look? | Agitated, swinging at people, and yelling. Trying to get out of bed and not responding to redirection. Pulled out IV; we were able to stop the bleeding. |
| Any new medications? | IV fluids since admission. Tylenol and oxycodone for pain. |
| What was her behavior like earlier today? | Per day nurse, she was pleasant with no agitation. |
| Is she at risk of harming herself or others? | Yes, I am worried she is going to fall out of bed and hurt herself. I’m also worried about our staff’s safety because she is swinging at us. |
| Is she in pain? | She hasn’t complained of pain during my shift. Not answering questions currently. |
| Recent ECG? | Earlier today. Normal sinus rhythm. QTc 420 ms. |

**If intern states plan to evaluate patient in person, state:** “Can we get something now? I’m worried she is going to hurt herself or someone else. What about restraints?”

**If intern orders a PRN medication, state:** “When should I give this? When should I notify you again?”

**If intern orders restraints,** **state**: “Are you going to evaluate the patient before I put these on?”

**Page 3 – Maria RN**

**Page:** Smith F4/424. New red rash, stopped vancomycin. Doesn’t look like hives. Next steps? Maria.

**When intern calls, answer phone with**: “Unit front desk, how can I help you?” Then answer the phone as Maria.

**When intern specifies why they are calling, state:** “I noticed she had a red rash on her face and upper chest so I stopped her vancomycin. Do you want me to restart it?”

**If asked about specifically, the following should be provided.** If student asks for information you don’t have, state “I do not have that information.”

| One-Liner | 32F with obesity, DM2 on insulin, and Crohn’s disease on infliximab who was admitted 1 day ago with cellulitis of the left hand. |
| --- | --- |
| Vitals | T 99.1 F  BP 123/78  HR 65  RR 14  SpO2 98% on room air |
| Access | 1 PIV |
| Assessment/How does patient look? | Well-appearing. Alert and oriented. Breathing comfortably. Itchy from rash. Diffuse redness and flushing on face and upper chest. No hives. |
| Shortness of breath? | No |
| Chest pain? | No |
| Lip or tongue swelling? | No |
| GI symptoms (vomiting, abdominal pain)? | No |
| Allergies? | None listed |
| Any other new meds? | No other meds are running. Received zosyn earlier today. |
| When did vancomycin start? | Received a few doses over the past 24 hours. No reactions with previous doses. |
| Any change after stopping vancomycin? | Redness is starting to go away. Patient already feeling better. |
| MRSA swab? | Just came back negative |

**If intern does not ask about anaphylaxis symptoms, ask:** “Do you think this could be anaphylaxis? I haven’t seen it before but worry because this rash seems to be related to the vancomycin I gave her.”

**If intern requests epinephrine, state**: “I don’t feel comfortable giving that without the doctor at the bedside. Can you come see the patient?”

**If intern states plan to see the patient:** “Thanks! If it takes you a while, what should I be looking out for?”**Page 4 – Abby RN**

**Page:** Gates B6/638. K 3.3, replete? Thanks! Abby.

**When intern calls, answer phone with**: “Unit front desk, how can I help you?” Then answer the phone as Abby.

**When intern specifies why they are calling, state:** “I saw the K was low at 3.3. Do you want to order some repletion?”

**If asked about specifically, the following should be provided.** If student asks for information you don’t have, state “I do not have that information.”

| One-Liner | 59M with HFpEF (EF 55%), AS s/p TAVR, HTN, HLD, pAF on eliquis, and DM2 who was admitted yesterday with CHF exacerbation undergoing diuresis. |
| --- | --- |
| Vitals | T 98.6 F  BP 142/91  HR 67  RR 12  SpO2 94% on 4L |
| Assessment/How does he look? | Comfortable. Alert and oriented. No complaints. Breathing normally. |
| Labs earlier today? | Cr 1.1, K 3.0, Mg 2.0 |
| Magnesium? | They didn’t check that, only K. |
| Can he take oral potassium? | Yes |

**If intern states plan to order potassium, state:** “Thank you! I just know we’ve been diuresing him so thought you might want to order potassium.”**Page 5 – Ellen RN**

**Page:** Park F6/578. FYI temperature 102.6F. Ellen.

**When intern calls, answer phone with**: “Unit front desk, how can I help you?” Then answer the phone as Ellen.

**When intern specifies why they are calling, state:** “I paged because I wanted to let you know his temperature was above the notify parameters.”

“Can he also get something more for pain?”

**If asked about specifically, the following should be provided.** If student asks for information you don’t have, state “I do not have that information.”

| One-Liner | 25M with anxiety and alcohol use disorder who was admitted 2 days ago with abdominal pain due to pancreatitis. |
| --- | --- |
| Vitals | T 102.6F  BP 110/78  HR 111  RR 22  SpO2 94% on room air |
| Assessment/How does he look? | Looks like he’s in pain. Abdomen is tender but soft. Sweating. Lungs are clear. Alert and oriented. |
| Is he symptomatic? | Yes, he has worsening abdominal pain and feels hot and sweaty. Nauseated, vomited once. |
| What were his vital signs before? | T 98.0 F  BP 137/90  HR 90  RR 18  SpO2 94% on room air |
| Access? | 1 PIV |
| Details about abdominal pain? | Epigastric, radiating to back. Worse than earlier in the day. |
| Current meds? | Oxycodone, hydromorphone, acetaminophen, subcutaneous heparin, ondansetron |

**If intern states plan to come to the bedside, ask:** “What do you think is going on? Do you want to order anything right now?”

**If asked if you have any questions, ask:** “How often should I check vital signs? Do you think he needs a higher level of care? Are you going to order anything?”
