# Supplementary material for: Development and Implementation of an OSCE for Formative Assessment of Core Clinical Skills in Internal Medicine Interns
Source: MedEdPORTAL. 2026 Feb 20;22:11576. doi: 10.15766/mep_2374-8265.11576 (PMC12920606; doi:10.15766/mep_2374-8265.11576)
Supplement: Supplementary file 1 — Prebrief Guide.docxStation A - GI Case Instructions.docxStation A - ID Case Instructions.docxStation A - GI Facilitator Guide.docxStation A - ID Facilitator Guide.docxStation B - Instructions.docxStation B - SP Case.docxStation B - SP Guide.docxStation C - Instructions.docxStation C - Sign-Out Template.docxStation C - Facilitator Guide.docxStation D - Instructions.docxStation D - Orders Form.docxStation D - Facilitator Guide.docxStation D - Page Delivery Instructions.docxStation A - Evaluator Checklist.docxStation B - Evaluator Checklist.docxStation C - Evaluator Checklist.docxStation D - Evaluator Checklist.docxPre- and Postsurveys.docx [file mep_2374-8265.11576-s001.zip › O. Station D - Page Delivery Instructions.docx]

**Appendix O: Station D – Paging**

**Page Delivery Instructions**

**Set #1**: At the station's beginning, send all 3 pages simultaneously to the two pagers. The beginning of the station will be announced overhead.

Page 1: Wallace F6/562. I just noticed this patient hasn’t had a bowel movement in 3 days. Can we get a PRN? Jen.

Page 2: Bolt B6/412. Patient with increasing agitation, pulled out IV. Can you order something? Please call Sara.

Page 3: Smith F4/424. New red rash, stopped vancomycin. Doesn’t look like hives. Next steps? Maria.

**Set #2:** After 8 minutes have elapsed, send the following 2 pages simultaneously to the two pagers.

Page 4: Gates B6/638. K 3.3, replete? Thanks! Abby.

Page 5: Park F6/578. FYI temperature 102.6F. Ellen.
