# Supplementary material for: Development and Implementation of an OSCE for Formative Assessment of Core Clinical Skills in Internal Medicine Interns
Source: MedEdPORTAL. 2026 Feb 20;22:11576. doi: 10.15766/mep_2374-8265.11576 (PMC12920606; doi:10.15766/mep_2374-8265.11576)
Supplement: Supplementary file 1 — Prebrief Guide.docxStation A - GI Case Instructions.docxStation A - ID Case Instructions.docxStation A - GI Facilitator Guide.docxStation A - ID Facilitator Guide.docxStation B - Instructions.docxStation B - SP Case.docxStation B - SP Guide.docxStation C - Instructions.docxStation C - Sign-Out Template.docxStation C - Facilitator Guide.docxStation D - Instructions.docxStation D - Orders Form.docxStation D - Facilitator Guide.docxStation D - Page Delivery Instructions.docxStation A - Evaluator Checklist.docxStation B - Evaluator Checklist.docxStation C - Evaluator Checklist.docxStation D - Evaluator Checklist.docxPre- and Postsurveys.docx [file mep_2374-8265.11576-s001.zip › P. Station A - Evaluator Checklist.docx]

**Appendix P: Station A – Calling a Consult**

**Evaluator Instructions and Checklist**

You will observe an intern reading clinical notes and calling a fellow to request a consult. The intern should provide a succinct summary of the case and a clear question. The call-back number is listed in the room near the phone, and the intern should place the phone on speaker. Please complete the checklist while observing this conversation.

At the end, there will be 5 minutes to provide immediate verbal feedback on areas performed well and constructive feedback on areas for improvement. Please allow the fellow to provide feedback first. If you think it would be helpful to share with the intern's coach, please note pertinent input from the fellow in the comments area of the checklist.

**Intern OSCE Station A: Calling a Consult** Intern Name___________________________________**_**

| **5 Cs Model Checklist for Assessing Physician Consultations** | | | |
| --- | --- | --- | --- |
| The five Cs and explanations | Checklist item | Done | Not done |
| *Contact*  Introducing the consulting and consultant physicians. Building the relationship. | 1. States name 2. States rank and service 3. Identifies supervising attending 4. Identifies name of consultant physician | **□**  **□**  **□**  **□** | **□**  **□**  **□**  **□** |
| *Communicate*  Giving a concise story and asking focused questions. | 1. Presents a concise story 2. Presents an accurate recount of information/case detail 3. Speaks clearly | **□**  **□**  **□** | **□**  **□**  **□** |
| *Core question*  Preparing a specific question for request of the consultant. Deciding on reasonable timeframe for consultation. | 1. Specifies need for consultation. 2. Specifies timeframe for consultation. | **□**  **□** | **□**  **□** |
| *Collaboration*  Planning a course of action that results from the discussion between the consulting physician and the consultant, including any alteration of management or testing. | 1. Is open to and incorporates consultant’s recommendations. | **□** | **□** |
| *Closing the loop*  Ensuring that both parties agree to the plan and to maintaining proper communication about any changes in the patient’s status. | 1. Reviews and repeats patient care plan. 2. Thanks consultant for consultation. | **□**  **□** | **□**  **□** |

| **Global Rating Scale (GRS) for Assessing Physician Consultations** | | | | | |
| --- | --- | --- | --- | --- | --- |
| Performance characteristic | Rating | | | | |
|  | Not effective | Somewhat effective | Effective | Very effective | Extremely effective |
| Introduction of involved parties |  |  |  |  |  |
| Patient case presentation |  |  |  |  |  |
| Specified consultation objective |  |  |  |  |  |
| Case discussion |  |  |  |  |  |
| Confirmation and closing |  |  |  |  |  |
| Interpersonal skills |  |  |  |  |  |
| Global rating |  |  |  |  |  |

Comments:
