# Supplementary material for: Development and Implementation of an OSCE for Formative Assessment of Core Clinical Skills in Internal Medicine Interns
Source: MedEdPORTAL. 2026 Feb 20;22:11576. doi: 10.15766/mep_2374-8265.11576 (PMC12920606; doi:10.15766/mep_2374-8265.11576)
Supplement: Supplementary file 1 — Prebrief Guide.docxStation A - GI Case Instructions.docxStation A - ID Case Instructions.docxStation A - GI Facilitator Guide.docxStation A - ID Facilitator Guide.docxStation B - Instructions.docxStation B - SP Case.docxStation B - SP Guide.docxStation C - Instructions.docxStation C - Sign-Out Template.docxStation C - Facilitator Guide.docxStation D - Instructions.docxStation D - Orders Form.docxStation D - Facilitator Guide.docxStation D - Page Delivery Instructions.docxStation A - Evaluator Checklist.docxStation B - Evaluator Checklist.docxStation C - Evaluator Checklist.docxStation D - Evaluator Checklist.docxPre- and Postsurveys.docx [file mep_2374-8265.11576-s001.zip › Q. Station B - Evaluator Checklist.docx]

**Appendix Q: Station B – Informed Consent**

**Evaluator Instructions and Checklist**

You will observe an intern obtaining informed consent for a blood transfusion with a standardized patient. The intern should discuss the benefits and risks of blood transfusion and alternative therapies. Please complete the checklist while observing this patient encounter.

At the end, there will be 5 minutes to provide immediate verbal feedback on areas performed well and constructive feedback on areas for improvement. Please allow the standardized patient to provide feedback first. If you think input from the standardized patient would be helpful to share with the intern’s coach, please include it in the comments area of the checklist.

At the end of the case, please collect the signed consent form.

**Intern OSCE Station B: Informed Consent** Intern Name ­___________________________________**_**

| **Topic** | **Done** | **Not Done** | **Feedback** |
| --- | --- | --- | --- |
| 1. Introduces themselves & identifies their role as cross-cover provider. | **□** | **□** |  |
| 2. Intern discusses the anemia due to GI blood loss and recommendation for blood transfusion. | **□** | **□** |  |
| 3. Intern discusses the risk of blood transfusion  a. Allergic reaction  b. Infection transmission  c. Other (e.g., TACO, TRALI, fever, etc.) | **□** | **□** |  |
| 4. Intern discusses benefits of transfusion (ex: oxygen delivery, function of organs, etc) | **□** | **□** |  |
| 5. Intern discusses no transfusion as an option, and what the plan would be. | **□** | **□** |  |
| 6. Intern discusses alternates to transfusion (e.g. fluids, IV iron) | **□** | **□** |  |
| 7. Intern checks for understanding from the patient. Asks if they have questions. | **□** | **□** |  |
| 8. Intern has patient sign consent form. | **□** | **□** |  |
| 9. Any general feedback on communication style? (Eye contact, jargon use, etc.) | **□** | **□** |  |

Comments:
