# Supplementary material for: Development and Implementation of an OSCE for Formative Assessment of Core Clinical Skills in Internal Medicine Interns
Source: MedEdPORTAL. 2026 Feb 20;22:11576. doi: 10.15766/mep_2374-8265.11576 (PMC12920606; doi:10.15766/mep_2374-8265.11576)
Supplement: Supplementary file 1 — Prebrief Guide.docxStation A - GI Case Instructions.docxStation A - ID Case Instructions.docxStation A - GI Facilitator Guide.docxStation A - ID Facilitator Guide.docxStation B - Instructions.docxStation B - SP Case.docxStation B - SP Guide.docxStation C - Instructions.docxStation C - Sign-Out Template.docxStation C - Facilitator Guide.docxStation D - Instructions.docxStation D - Orders Form.docxStation D - Facilitator Guide.docxStation D - Page Delivery Instructions.docxStation A - Evaluator Checklist.docxStation B - Evaluator Checklist.docxStation C - Evaluator Checklist.docxStation D - Evaluator Checklist.docxPre- and Postsurveys.docx [file mep_2374-8265.11576-s001.zip › R. Station C - Evaluator Checklist.docx]

**Appendix R: Station C – Sign-Out**

**Evaluator Instructions and Checklist**

You will observe an intern providing sign-out on 2 patients to the cross-covering senior resident. The intern will have 10 minutes to read the cases (an H&P and progress note) and write sign-out. Then, a senior resident will enter the room, and you will observe the intern provide verbal sign-out to the resident (~5 minutes) while you complete the faculty observation form.

There will be 5 minutes to provide immediate verbal feedback on areas performed well and constructive feedback on areas for improvement. Please allow the resident to provide feedback first. If you think input from the senior resident would be helpful to share with the intern’s coach, please include it in the comments area of the checklist.

At the end of the case, please save the written Word document on the desktop and ensure the intern’s name is written at the top of the document. The intern will receive feedback on their written documentation during the meeting with their coach.

**Sign-Out Direct Observation (adapted from I-PASS Study)**

**Observer Information:**Name: Date: __/__/__

**Resident Information:**

Name: __________________________

**1. Was a tailored summary provided by the resident giving the handoff (e.g. description of the “big picture” of what will need to be prioritized by the receivers of the handoff):**

**⁯** Yes

**⁯** No

**2.** **Indicate which specific elements of the mnemonic were used with each handoff**

Case 1

| **Verbal Mnemonic** | **Description** | **No** | **Partially** | **Yes** |
| --- | --- | --- | --- | --- |
| **Illness Severity** | Identification as stable, “watcher”, or unstable |  |  |  |
| **Patient Summary** | Summary statement, events leading up to admission, hospital course, ongoing assessment, plan |  |  |  |
| **Action List** | To-do list; timeline and ownership |  |  |  |
| **Situation Awareness/Contingency Planning** | Know what’s going on; plan for what might happen |  |  |  |
| **Synthesis by Receiver** | Ensures receiver summarizes what was heard, asks questions, restates key action/to do items |  |  |  |

Case 2

| **Verbal Mnemonic** | **Description** | **No** | **Partially** | **Yes** |
| --- | --- | --- | --- | --- |
| **Illness Severity** | Identification as stable, “watcher”, or unstable |  |  |  |
| **Patient Summary** | Summary statement, events leading up to admission, hospital course, ongoing assessment, plan |  |  |  |
| **Action List** | To do list; timeline and ownership |  |  |  |
| **Situation Awareness/Contingency Planning** | Know what’s going on; plan for what might happen |  |  |  |
| **Synthesis by Receiver** | Ensures receiver summarizes what was heard, asks questions, restates key action/to do items |  |  |  |

**Rate the frequency with which the resident who gave the handoff did the following:**3. Actively engages receiver to ensure shared understanding of patients (Encouraged questions, asked questions)

**⁯** Never **⁯** Rarely **⁯** Sometimes **⁯** Often**⁯** Always

4. Appropriately prioritizes key information, concerns, or actions

**⁯** Never **⁯** Rarely **⁯** Sometimes **⁯** Often **⁯** Always

**Rate the frequency with which the resident who gave the handoff did the following:**5. Miscommunications or transfer of erroneous information

**⁯** Never **⁯** Rarely **⁯** Sometimes **⁯** Often **⁯** Always

6. Omissions of important information

**⁯** Never **⁯** Rarely **⁯** Sometimes **⁯** Often **⁯** Always

7. Tangential or unrelated conversation

**⁯** Never **⁯** Rarely **⁯** Sometimes **⁯** Often **⁯** Always

**8. Rate your overall impression of the *pace* of the handoff:**

⁮ Very slow pace/ Very inefficient

⁮Slow pace/ Inefficient

⁮Optimally paced/ Efficient but not rushed

⁮ Fast/pressured pace

⁮Very fast/pressured pace

**9. What was especially effective about the handoff?**

**10. What aspect(s) of the handoff could be improved?**

**11. Did you provide the resident feedback regarding their handoff?**

**⁯** Yes

**⁯** No
