# Supplementary material for: Development and Implementation of an OSCE for Formative Assessment of Core Clinical Skills in Internal Medicine Interns
Source: MedEdPORTAL. 2026 Feb 20;22:11576. doi: 10.15766/mep_2374-8265.11576 (PMC12920606; doi:10.15766/mep_2374-8265.11576)
Supplement: Supplementary file 1 — Prebrief Guide.docxStation A - GI Case Instructions.docxStation A - ID Case Instructions.docxStation A - GI Facilitator Guide.docxStation A - ID Facilitator Guide.docxStation B - Instructions.docxStation B - SP Case.docxStation B - SP Guide.docxStation C - Instructions.docxStation C - Sign-Out Template.docxStation C - Facilitator Guide.docxStation D - Instructions.docxStation D - Orders Form.docxStation D - Facilitator Guide.docxStation D - Page Delivery Instructions.docxStation A - Evaluator Checklist.docxStation B - Evaluator Checklist.docxStation C - Evaluator Checklist.docxStation D - Evaluator Checklist.docxPre- and Postsurveys.docx [file mep_2374-8265.11576-s001.zip › S. Station D - Evaluator Checklist.docx]

**Appendix S: Station D – Paging**

**Evaluator Instructions and Checklist**

You will observe the intern receiving multiple pages and calling back the nurse to address their concerns. The intern will receive two batches of pages: Pages 1-3 at the beginning of the station and Pages 4-5 after 8 minutes have elapsed.

They may not have time to call back every page and must triage the higher priority pages to call back first. If the intern does not call back the nurse (there are some pages for which a call back is unnecessary), they may write orders on a printed orders form in the room. The call-back number is listed in the room near the phone and is the same for each page. The intern should place the phone on speaker. You will observe the conversations and complete the checklist.

After 15 minutes have elapsed, there will be 5 minutes to provide immediate verbal feedback on areas performed well and constructive feedback on areas for improvement. Please allow the nurse to provide feedback first. If the intern is no longer on the phone with the nurse, please instruct the intern to call them back. If you think it would be helpful to share with the intern's coach, please include pertinent input from the nurse in the comments area of the checklist.

At the end of the session, **please delete the pages on the pager and collect the paper order form.**

**Intern OSCE Station D: Paging** Intern Name ­_____________________

| **Task** | **Yes** | **Partially** | **No** |
| --- | --- | --- | --- |
| Triages pages appropriately, calling back higher priority requests first |  |  |  |
| Introduces self and identifies reason for calling |  |  |  |
| Asks specific, goal-directed questions |  |  |  |
| Indicates plan to evaluate the patient in person when appropriate |  |  |  |
| Provides a preliminary plan |  |  |  |
| Explains their thought process |  |  |  |
| Asks if RN has additional questions before concluding call |  |  |  |
| Uses closed-loop communication |  |  |  |
| Writes orders if does not call nurse back for lower priority requests |  |  |  |

**Page 1:** Low Priority

Page: Wallace F6/562. I just noticed this patient hasn’t had a bowel movement in 3 days. Can we get a PRN? Jen.

| **Task** | **Yes** | **Partially** | **No** |
| --- | --- | --- | --- |
| Orders or states plan to order a bowel regimen |  |  |  |

Comments:

**Page 2:** High priority

Page: Bolt B6/412. Patient with increasing agitation, pulled out IV. Can you order something? Please call Sara.

| **Task** | **Yes** | **Partially** | **No** |
| --- | --- | --- | --- |
| Assesses degree of agitation: does nurse feel patient is a danger to self or others? |  |  |  |
| States plan to evaluate patient in person |  |  |  |
| Does not order lorazepam |  |  |  |
| Explains thought process for not ordering lorazepam |  |  |  |
| If orders medication, explains to nurse when to give it and when to notify again |  |  |  |
| Demonstrates empathy when nurse expresses concern and frustration |  |  |  |

Comments:

**Page 3:** Medium priority

Page: Smith F4/424. New red rash, stopped vancomycin. Doesn’t look like hives. Next steps? Maria.

| **Task** | **Yes** | **Partially** | **No** |
| --- | --- | --- | --- |
| Asks for description of rash |  |  |  |
| Requests vital signs |  |  |  |
| Asks about signs and symptoms of anaphylaxis |  |  |  |
| States plan to evaluate patient in person |  |  |  |
| Instructs nurse to not restart vancomycin |  |  |  |
| Provides instructions to nurse for what clinical signs to watch out for and when to call again |  |  |  |

Comments:

**Page 4:** Low priority

Page: Gates B6/638. K 3.3, replete? Thanks! Abby.

| **Task** | **Yes** | **Partially** | **No** |
| --- | --- | --- | --- |
| Orders or states plan to order potassium repletion |  |  |  |

Comments:

**Page 5:** High priority

Page: Park F6/578. FYI temperature 102.6F. Ellen.

| **Task** | **Yes** | **Partially** | **No** |
| --- | --- | --- | --- |
| Requests other vital signs |  |  |  |
| States plan to evaluate patient in person |  |  |  |
| Identifies concern for sepsis |  |  |  |
| Orders or states plan to order blood cultures and labs |  |  |  |
| Provides instructions to RN regarding frequency of monitoring and level of care |  |  |  |

Comments:

**Overall Comments:**
